# Neem Leaf Glycoprotein Restrains VEGF Production by Direct Modulation of HIF1α-Linked Upstream and Downstream Cascades

**DOI:** 10.3389/fonc.2020.00260

**Published:** 2020-03-06

**Authors:** Akata Saha, Partha Nandi, Shayani Dasgupta, Avishek Bhuniya, Nilanjan Ganguly, Tithi Ghosh, Ipsita Guha, Saptak Banerjee, Rathindranath Baral, Anamika Bose

**Affiliations:** Department of Immunoregulation and Immunodiagnostics, Chittaranjan National Cancer Institute (CNCI), Kolkata, India

**Keywords:** hypoxia, VEGF, HIF1α, STAT3, tumor-microenvironment, NLGP

## Abstract

Neem Leaf Glycoprotein (NLGP) is a natural immunomodulator, have shown sustained tumor growth restriction as well as angiogenic normalization chiefly by activating CD8^+^ T cells. Here, we have investigated the direct role of NLGP as a regulator of tumor microenvironmental hypoxia and associated vascular endothelial growth factor (VEGF) production. We observed a significant reduction in VEGF level in both *in vivo* murine tumor and *in vitro* cancer cells (B16Mel, LLC) and macrophages after NLGP treatment. Interestingly, NLGP mediated VEGF downregulation in tumor cells or macrophages within hypoxic chamber was found at an early 4 h and again at late 24 h in mRNA level. Our data suggested that NLGP prevented hypoxia-induced strong binding of HIF1α with its co-factors, CBP/p300 and Sp3, but not with Sp1, which eventually limit the binding of HIF1α-transcriptional complex to hypoxia responsive element of VEGF promoter and results in restricted early VEGF transcription. On the otherhand, suppressed phosphorylation of Stat3 by NLGP results reduction of HIF1α at 24 h of hypoxia that further support sustained VEGF down-regulation. However, NLGP fails to regulate VHL activity as observed by both *in vivo* and *in vitro* studies. Therefore, this study for the first time reveals a mechanistic insight of NLGP mediated inhibition of angiogenesis by suppressing VEGF, which might help in vascular normalization to influence better drug delivery.

## Summary

NLGP downregulates VEGF at 4 h transcriptionally and both VEGF and HIF1α at 18 h translationally. Co-factors of HIF1α-transcriptional-complex downregulated at 4 h in hypoxia by NLGP. Downregulation of pStat3 by NLGP downregulates HIF1α resulting in a sustained VEGF reduction in hypoxia.

## Introduction

Malignant solid tumor progression needs aberrant angiogenic stimuli for rapid neovascularization to maintain nutrient and oxygen supply to proceed beyond 2 mm^3^ (1, 2). Among various pro-angiogenic factors, vascular endothelial growth factor (VEGF) is the most influential factor responsible for promoting tumor angiogenesis despite the nature and origin of tumor. Thus, VEGF targeting has efficacy in several cancer management (3, 4). Rapid tumor cell proliferation creates hypoxia or oxygen deficit (5) and stabilizes HIF1α, the main VEGF regulator along with growth factors and oncogenes (6, 7). Within the tumor microenvironment (TME), tumor cells themselves along with non-tumor stromal cells serves as the source of VEGF (8).

HIFs are basic helix-loop-helix transcription factors belonging to the PAS (PER/aryl hydrocarbon receptor nuclear translocator) family. It has two subunits, an oxygen-sensitive α-subunit and a constitutively expressed β-subunit (9). In normoxia, α-subunit undergoes post-transcriptional modifications with prolyl-hydroxylase (PHD) and von-Hippel-Lindau (VHL) leading to proteosomal degradation via E3 ubiquitin ligase (10). However, in hypoxia, HIF1α becomes stable and translocate to the nucleus where it forms a heterodimer with HIF1β and many other co-factors to activate more than 124 significant genes (11). In addition to hypoxia, several other factors are potent stimulator of either HIF1α or VEGF or both. Growth factors, cytokines etc. by increase HIF1α protein synthesis via activation of PI3K/AKT (12) or ERK/MAPK pathways (13) or STAT3 signaling pathway (14, 15). Interestingly, recent work suggested a co-operative role of STAT3 and HIF1α in VEGF upregulation (16). VEGF expression can also be induced by HIF1α independent manner by STAT3, AP1, Sp1, and cAMP etc. (16, 17).

Targeting VEGF by many agents, including anti-VEGF monoclonal antibodies, have been developed showing promising effect *in vitro*, but their effects *in vivo* settings or in cancer patients are limited due several adverse effects, such as hypertension, gastrointestinal-perforation, bleeding, impairment of wound healing etc. (18). On the other hand, several plant based natural molecules or anti-oxidants show promises in reducing VEGF but their mechanisms are largely unknown. Neem leaf glycoprotein (NLGP), a non-toxic immune-modulator, show sustained tumor growth restriction in multiple murine cancer settings primarily by activating CD8^+^ cytotoxic T cells (19, 20). We also reported normalization of aberrant angiogenesis in murine carcinoma and melanoma hosts in an immune dependent manner (21). Therefore, this is of immense interest to study whether and how NLGP restricts VEGF synthesis and secretion from tumor resident cells.

Herein, we show that NLGP primarily targets VEGF synthesis by disrupting the binding of HIF1α with its co-factors, which ultimately prevents binding of HIF1α- transcriptional complex to the HRE region of VEGF. Additionally, NLGP prevents Stat3 activation and STAT3-dependent HIF1α transcription. Both of these events simultaneously mitigate VEGF secretion from tumor and non-tumor stromal cells.

## Materials and Methods

### Antibodies and Reagents

DMEM-high glucose medium and Fetal bovine serum (FBS) were obtained from Invitrogen (NY, USA). Purified anti-mouse antibodies (VEGF, HIF1α, Sp1, Sp3, p300, CBP, pAKT, pERK, STAT3, pSTAT3) and Stat3 siRNA were procured from Santa Cruz Biotechnology (Dallas, TX, USA). Anti-mouse/rabbit fluorescence conjugated secondary antibodies (FITC and PE conjugate) were purchased from Sigma Aldrich (St. Louis, US). RT-PCR primers were designed and procured from Eurofins, Bangalore, India. Trizol reagent for RNA isolation and Revert Aid™ cDNA synthesis kit were procured from Invitrogen (Carlsbad, CA, USA) and Fermentas (Waltham, MA, USA), respectively.

### Maintenance of Cell Lines

B16F10 murine melanoma cells (B16Mel) were purchased from the National Center for Cell Sciences (NCCS), Pune, India. Lewis Lung Carcinoma (LL/2 (LLC1) were purchased directly from American Type Cell Culture (ATCC® CRL1642™, Manassas, VA, USA). Macrophages were collected from peritoneal cavity of C57BL/6J mice and tumor conditioned using B16Mel tumor lysate. Cells were maintained at 70% confluency in complete DMEM high glucose media supplemented with 10% (v/v) heat inactivated FBS, 2mM L-glutamine, 100 U/ml penicillin and 100 μg/ml streptomycin at 37°C with the supply of 5% CO_2_. Authentication is done using STR method in the cell banks. All cells were maintained for 10 to 12 passages and all handling procedure was done according to guidelines provided by ATCC. B16Mel cells were tested for mycoplasma contamination using mycoplasma detection kit (EZdetect™ PCR Kit for Mycoplasma Detection; based on 16s-23s rRNA spacer region, Himedia, India). All experiments were done within 6 months of purchase.

### Mice and Tumor Inoculation

Inbred female C57BL/6J mice (age, 4–6 weeks, average body weight 21 g) were obtained and maintained as described (21). All experiments were performed in accordance with the guidelines provided by the Institutional Animal Care and Ethics Committee (Approval No. IAEC-1774/RB-7/2016/3).

### Neem Leaf Glycoprotein

Neem leaf glycoprotein (NLGP) was prepared from neem leaves (*Azadirachta indica*), by the method as described previously (21). A standard protocol was followed as described (22).

### Generation of Hypoxic Environment *in vitro*

B16Mel cells were kept in a hypoxia chamber (Stem Cell Technologies, Canada) to mimic artificial tumor hypoxic environment. The later was generated by passing a hypoxia gas mixture of 5% O_2_, 85% N_2_, and 10% CO_2_, at 20psi pressure for 4 min. According to manufacturer's protocol, this time is required to completely replace the atmospheric gas inside the chamber with the desired gas mixture.

### Histology and Immunohistochemistry

Tumors were fixed to stain with VEGF according to method described (19). In some cases, VEGF positive regions were selected and dissected using laser capture microscopy.

### Cytokine Detection Assay

Cytokines secreted from B16Mel cells within culture supernatant both in hypoxia and normoxia w/wo NLGP, were measured by ELISA as described (19).

### Co-localization Studies

B16Mel cells were grown in chamber slides in hypoxia, w/wo NLGP, fixed with paraformaldehyde and permeabilized in 0.1% Triton X-100. After methodical washing with PBS-Tween 20 and PBB (0.5% BSA + PBS) blocking was done using 2% BSA. Primary antibodies for Sp1, Sp3, CBP, p300 and HIF1α were added in dilution range 1:200 to 1:500 and incubated in a moist chamber, overnight. Secondary antibody was added and incubated for 1 h. Slides were finally mounted with DAPI and images were acquired using Leica DM 1000, Fluorescent Microscope (Leica, BM 4000B, Germany).

### Nuclear and Cytosolic Extraction

B16Mel cells were scraped using chilled PBS, centrifuged and cell pellet was re-suspended in ice-cold EMSA buffer to incubate for 1 h at 4°C. Nuclear and cytosolic fractions were carried out according to the protocol described (19).

### RT-PCR

Cellular RNA was isolated using Trizol (Invitrogen, Camarillo, CA) and random hexamers were used to generate corresponding cDNA (First Strand cDNA Synthesis Kit; Fermentas, Hanover, MD). Amplification was done according to protocol described (22).

### Co-immunoprecipitation and Western Blot

B16Mel cells were subjected to hypoxia w/wo NLGP for 4 h, and Co-IP of Sp1/3, CBP, p300 with HIF1α was performed as described (23).

### Chromatin Immunoprecipitation (ChIP) Assay

ChIP assays were conducted following the manufacturer's protocol (Millipore, Darmstadt, Germany). B16Mel cells (1 × 10^6^) were subjected to normoxia and hypoxia w/wo NLGP as described (23) was followed. DNA was extracted using phenol/chloroform to conduct PCR using promoter specific primers: HRE region in the promoter of VEGF (HIF1α binding site): sense 5′-CCACAGTGCATACGTGGGCTC-3′, antisense 5′-GGTGTCACGTATGCACCCGAG-3′.

### si-RNA Mediated STAT3 Silencing

STAT3 specific si-RNA (Santacruz Biotechnology, Dallas, TX) was added in 70% confluent B16Mel culture as described (23). Finally, expression of *stat3* and *hif1*α was checked both in untreated, NLGP treated and siRNA transfected B16Mel cells by RT-PCR.

### Flow-Cytometric Staining

Flow-cytometry of pAKT, pERK and pSTAT3 was done as described (24).

### Statistical Analysis

All reported results represent the mean ± SD of data obtained in either six (for *in vivo* analysis) or three to six (*in vitro* assays) independent experiments. Statistical significance was established by unpaired *t*-test using INSTAT 3 Software (Graphpad Inc., USA), with differences between groups attaining a *p*-value < 0.01 considered as significant.

## Results

### NLGP Mediated Tumor Growth Restriction Is Associated With Downregulation of VEGF in Immune and Tumor Cells

**Figure 1:**
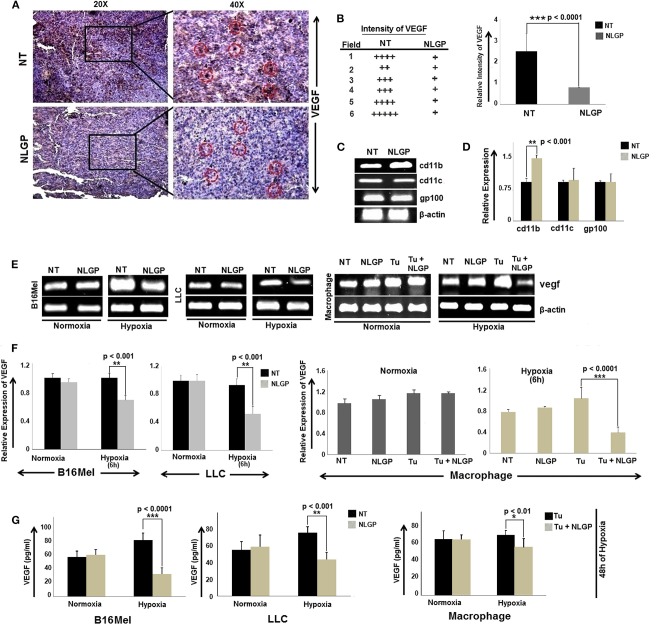
NLGP downregulates VEGF expression in tumor, cultured cancer cells and immune cells. **(A)** Immunohistochemical expression of VEGF in untreated and NLGP treated murine B16Mel tumor tissue. **(B)** Representative figures from tumor bearing (*n* = 6) hosts are presented in 20X and 40X magnification. Intensity of staining in six different fields from tumor sections of untreated and NLGP treated mice. Mean ± SD of staining intensity of VEGF are also shown. **(C)** Tumors from untreated and NLGP treated (1.5 μg/ml) C57BL6/J mice were cryosectioned and mRNA expression of *cd11c, cd11b, gp100* genes after laser capture microdissection of VEGF positive zones were measured using RT-PCR, keeping β-actin as loading control. **(D)** A bar diagram represents the mean ± SD of relative expression of respective genes (*n* = 4). **(E)** Expression of *vegf* gene is shown in B16Mel, LLC and macrophages cultured in both normoxic and hypoxic conditions (6 h of NLGP and hypoxia exposure) (*n* = 6 for each cell type), keeping β-actin as loading control. **(F)** Bar diagrammatic representation show mean ± SD of relative expression of vegf from all untreated and NLGP treated cell lines. **(G)** A bar diagram showing mean ± SD (*n* = 6) of VEGF levels as measured by ELISA in B16Mel, LLC and macrophage cells obtained from both hypoxic and normoxic conditions, w/wo NLGP. Cells were pre-treated for 48 h in NLGP and kept in 12 h hypoxia. *P*-values showing significance of difference are indicated in respective bar diagrams.

In continuation to our previous research in understanding the mechanism of NLGP mediated tumor growth restriction, we have reported that therapeutic NLGP treatment significantly reduces the availability of VEGF in tumor hosts, in an immune dependent (CD8^+^ T cells and IFNγ) manner (21). To analyze the detailed molecular mechanism behind NLGP mediated modulation of VEGF, here, first we studied the cellular source of VEGF, those are targeted by NLGP. Laser-capture-microdissection of tumor tissues followed by RT-PCR analysis suggested that *in vivo* NLGP treatment showed no change in melanoma (gp100) and dendritic cell (DC) (CD11c) marker, but an increase in macrophage marker (CD11b) (Figure 1C). Thus, observed reduction in VEGF as shown in Figure 1A might be due to NLGP's inhibition on VEGF secretion from B16Mel and macrophage cells (Figures 1A–D). This observation is suggestive that VEGF reduction is not due to direct killing of the tumor cells but rather NLGP could modulate tumor cells to restrict VEGF production.

Based on this *in vivo* result, next we studied VEGF expression of B16Mel and LLC cells and in macrophages under normoxia and hypoxia with NLGP treatment. RT-PCR and ELISA suggested a significant decrease in *vegf* gene as well as protein expression after 6 h and 48 h of NLGP treatment, respectively, in all cells studied. The extent of VEGF reduction was prominent under hypoxic condition with no change in normoxia (Figures 1E–G).

### NLGP Modulates HIF1α in Tumor Cells to Restrain VEGF Production

**Figure 2:**
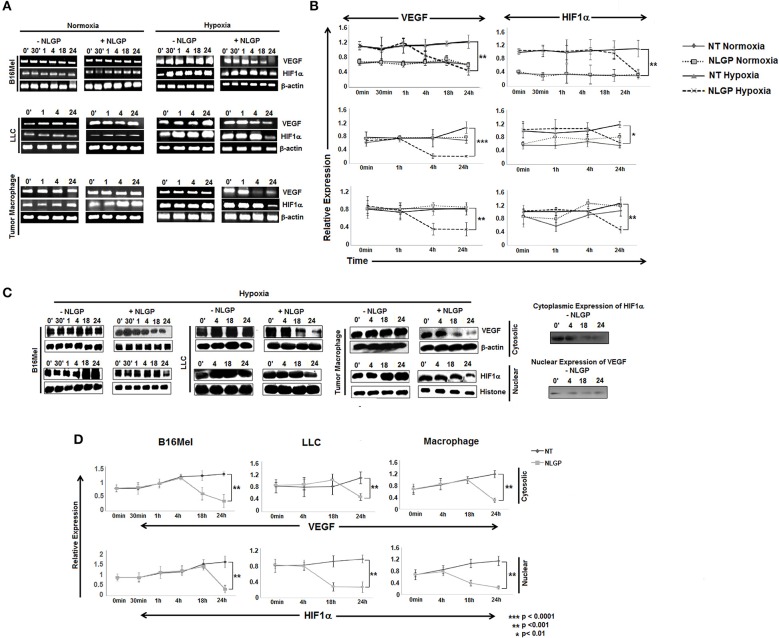
NLGP modulates HIF1α to restrain VEGF secretion in hypoxia. **(A)** B16Mel, LLC, and macrophages were treated w/wo NLGP and subjected to either hypoxia or kept in normoxia for different time periods. (A1) Representative data from *n* = 6 are shown, exhibiting mRNA for *vegf* and *hif1*α gene were studied by RT-PCR. β-actin was kept as a loading control. **(B)** A line graph showing different time points (0 min, 30 min, 1 h, 4 h, 18 h, and 24 h) represents the mean ± SD of relative expression of *vegf* and *hif1*α genes. *p*-value significance of hypoxia exposed sample are shown in all graphs, no significant change was observed in normoxic samples. **(C)** Western blotting was performed to check the protein level expression of VEGF and HIF1α in hypoxia for both untreated and NLGP treated cells at various time points. In the upper panel, cytosolic expression of VEGF is shown for various time points, β-actin was kept as loading control. Lower panel represents the nuclear expression of HIF1α keeping histone H3 as loading control. Basal level of cytosolic expression of HIF1α and nuclear VEGF expression in controlled condition is shown alongside the panel. **(D)** A line graph (*n* = 6) represents the mean ± SD of cytosolic VEGF and nuclear HIF1α expression w/wo NLGP treatment in hypoxia. *p*-values showing significance of difference are indicated in the figure.

Given the direct effect of NLGP in reduction of VEGF in hypoxic condition, next we checked the involvement of HIF1α, the main regulator of VEGF and also the exact time point at which VEGF is downregulated. B16Mel, LLC and macrophage cells were exposed to normoxic and hypoxic conditions for various time points (0 min, 30 min, 1 h, 4 h, 18 h, and 24 h) in presence or absence of NLGP (1.5μg/ml). RT-PCR analysis suggested downregulation of *vegf* started at 4 h and reached a maximum decrease at 24 h (Figures 2A,B), whereas downregulation of *hif1*α was observed only at 24 h. Western blot analysis suggested a downregulation of total VEGF started at 18 h of NLGP treatment under hypoxia (Figures 2C,D; Supplementary Material). As hypoxia stabilizes HIF1α protein and promotes its translocation to nucleus from cytoplasm, next we studied its expression in both cases. Although cytosolic HIF1α expression remains low in all the conditions but hypoxia increases HIF1α expression in nucleus of B16Mel cells, while, NLGP treatment reduced stable HIF1α expression at 24 h (Figures 2C,D). Therefore, collectively these data suggest that NLGP does not affect HIF1α expression in either protein or mRNA level at 4 h to control their VEGF production in tumor cells.

### NLGP Neither Affects Hypoxia-Induced HIF1α Stabilization nor O_2_/VHL-Dependent HIF1α Degradation

NLGP mediated downregulation of VEGF gene transcription was achieved after 4 h. However, NLGP is unable to reduce stable HIF1α expression in B16Mel cells at this time point, which was observed only at 24 h in both transcriptional and translational levels. This result clearly pointed out that NLGP treatment does not affect the HIF1α stabilization in hypoxic condition.

**Figure 3:**
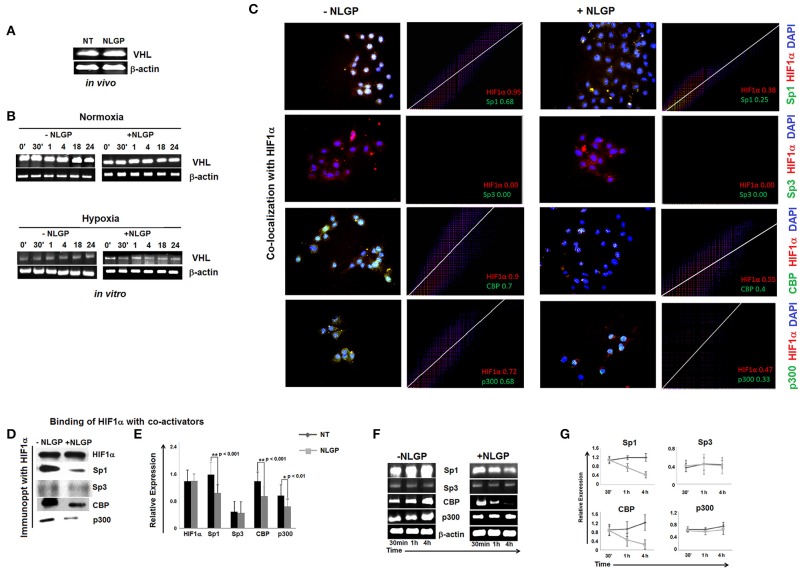
Effect of NLGP on VHL and HIF1α activational co-factors. **(A)** mRNA expression of tumor suppressor gene, *vhl* from both *in vivo* hosts **(A)** and *in vitro* B16Mel cells exposed to normoxia and hypoxia at different time points **(B)**, w/wo NLGP, was assessed by RT-PCR. β-actin was used as a loading control in all cases. **(C)** Co-localization of HIF1α and its co-activators in presence or absence of NLGP in hypoxia were determined by immunofluorescence microscopy and analyzed by *Image J* software as 2D histogram pictorial. Nuclei were stained with DAPI. HIF1α and co-activators, Sp1, Sp3, CBP, and p300 were detected with anti-mouse PE and anti-rabbit FITC, respectively. Representative data from *n* = 6 are presented. Mander's tM1 value greater than 0.6 indicates higher co-localization, tM1 value within 0.5–0.6 range indicates moderate co-localization and values below 0.5 indicates weak co-localization. tM1 values for each co-factors are mentioned in respective pictures. **(D)** Co-immunoprecipitation and western blot analysis of hypoxia (4 h) treated B16Mel cells from w/wo NLGP treatment as indicated. **(E)** A representative bar diagram showing mean ± SD from *n* = 6 are presented. **(F)** Representative data from *n* = 6 are shown, exhibiting mRNA expression using RT-PCR of *sp1, sp3, cbp*, and *p300* genes in hypoxia w/wo NLGP treatment at 3 different time points (30 min, 1 h, and 4 h). β-actin was used as a loading control. **(G)** A line diagram showing mean + SD (*n* = 6) representing change in expression with NLGP treatment form untreated samples are shown for three different time points (30 min, 1 h, and 4 h) of hypoxic condition.

Next, we look at VHL expression, an important tumor suppressor gene. In presence of O_2_, VHL ubiquinates HIF1α for its subsequent proteosomal degradation. However, neither *in vivo* (Figure 3A) nor *in vitro* (Figure 3B) NLGP treatment shows any effect in VHL expression in tumor tissues or in B16Mel cells, respectively, and suggested that NLGP mediated VEGF downregulation is not associated with VHL-dependent HIF1α degradation (Figures 3A,B).

### NLGP Targets the Binding of HIF1α to Its Co-activator and Prevents Formation of Active HIF1α Transcriptional Complex

HIF1α binds with HIF1β and other co-activators (like, Sp1, CBP/p300, etc.) to form active transcriptional complex after translocating to the nucleus and bind with hypoxia responsive elements (HRE) of VEGF promoter (25). As NLGP treatment does not show any effect on HIF1α stabilization, we assessed the binding of HIF1α with its co-activator(s). Accordingly, B16Mel cells were exposed to hypoxia w/wo NLGP (1.5 μg/ml) for 4 h and co-localization and co-immunoprecipitation assays were performed. Co-localization assay of B16Mel cells grown-on chamber slide in hypoxic environment demonstrated that NLGP significantly prevents co-localization of HIF1α with Sp1, CBP, and p300. Analysis of co-localization by Image J software of HIF1α with Sp1, CBP, and p300 shows higher Mander's tM1 value for all hypoxia treated cohorts (>0.6) compared to NLGP-treated cohorts (<0.5) (Figure 3C). Co-immunoprecipitation assay also supported that NLGP treatment is able to reduce protein-protein interaction of HIF1α with its co-activators (Figures 3D,E; Supplementary Material), whereas the binding between HIF1α and Sp3 (a competitive inhibitor of Sp1) showed no change in NLGP treated B16Mel cells (Figures 3C,D). Interestingly, NLGP treatment also downregulates transcriptional expression of both Sp1, CBP and p300 in B16Mel cells under hypoxic condition after 4 h (Figures 3F,G). Therefore, the overall data suggest that NLGP is able to prevent the formation of active HIF1α transcriptional complex required for VEGF transcription.

### NLGP Prevents Binding of HIF1α Transcriptional Complexes to VEGF Promoter in Hypoxia

**Figure 4:**
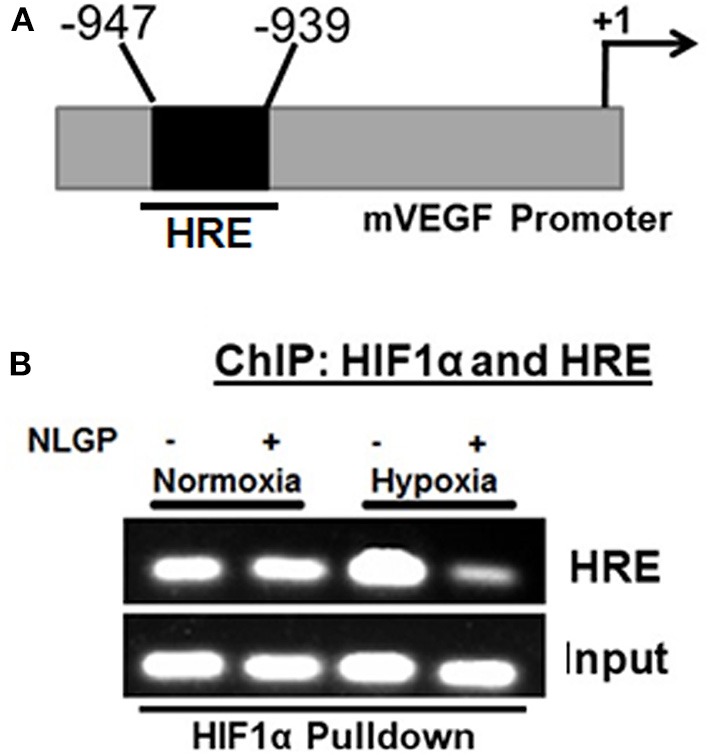
NLGP prevents nuclear binding of HIF complex to HRE. **(A)** Potential Hypoxia Responsive Element (HRE) within mouse *vegf* gene promoter. Indicated arrows are for PCR primer positions used in ChIP assay. **(B)** Representative data of mRNA expression using RT-PCR of HIF1α/co-activator complex recruitment to the HRE region of *vegf* promoter in NLGP treated or non-treated B16Mel cells in hypoxia. Input lanes are prepared from 10% of samples used in IPs, with control IgG and HIF1α activational complex.

As NLGP prevents the nuclear translocation of HIF1α and the formation of active HIF1α complex, next we assessed its binding with cognate HRE sequence at VEGF promoter using chromatin immune-precipitation (ChIP) assay. Results obtained from ChIP assay suggested that hypoxia remarkably promoted the binding of HIF1α with VEGF promoter in B16Mel cells whereas, NLGP treatment caused a significant reduction in binding of HIF1α with VEGF promoter in hypoxic condition (Figures 4A,B).

### NLGP Targets Stat3-Dependent Transcription of HIF1α to Reduce VEGF

**Figure 5:**
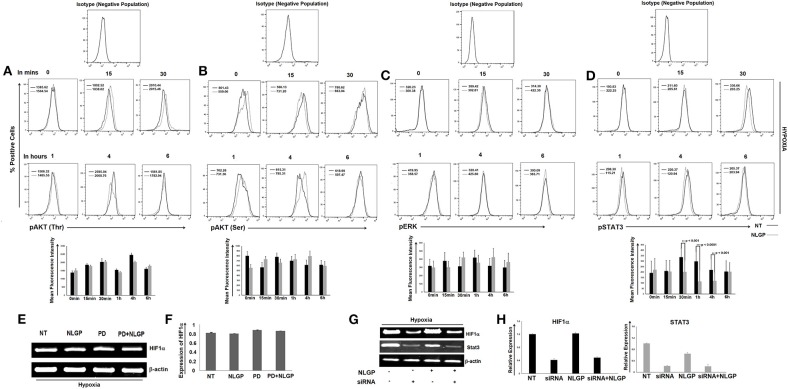
NLGP targets HIF1α upstream signaling cascade. **(A)** Responsible factors for regulating HIF1α at protein level was determined by a time kinetics experiment. B16Mel cells were exposed to hypoxia w/wo NLGP treatment for various time points (0 min, 15 min, 30 min, 1 h, 4 h, and 6 h) and expression pAKT(Thr) **(A)**, pAKT(Ser) **(B)**, pERK **(C)**, and pSTAT3 **(D)** was checked by flow cytometry. Illustrative histograms show percent positive cells for respective upstream signaling molecules (*n* = 6). Mean Fluorescence Intensity (MFI) measuring the mean level of phosphorylation is indicated in each histogram with a bar diagram showing mean + SD of *n* = 4 samples. **(E)** mRNA expression of *hif1*α gene after blocking with inhibitor PD was checked w/wo NLGP treatment, keeping β-actin as loading control. **(F)** Representative data and bar diagram showing mean ± SD (*n* = 6) of HIF1α expression by RT-PCR are presented. **(G)** B16Mel cells were either treated with NLGP or Stat3 siRNA or both in presence of hypoxia. mRNA expression by RT-PCR was checked for *hif1*α gene expression. Level of Stat3 was also checked to see efficacy of the silencing experiment. **(H)** Representative bar diagram showing mean ± SD of relative expression (*n* = 6) for respective genes are presented.

Although NLGP treatment downregulates VEGF primarily by targeting HIF1α binding with VEGF promoter, it also causes a significant downregulation of HIF1α in transcription level at 24 h. Several transcription factors or oncogenic stimuli are reported to have HIF1α promoting activity like Stat3, NF-κB, AKT, ERK etc. To study the target molecule(s) in our settings, we have checked the activation status of different HIF1α-linked up-stream transcription factors in NLGP-treated B16Mel cells w/wo hypoxia by flow-cytometry and western blot analysis. Both the assays suggested that after 30 min of NLGP treatment there was a significant downregulation of pStat3 expression, which further showed a decrease at 1 h and maintained a reduced pStat3 expression till 4 h in hypoxia-exposed tumor cells (Figures 5A–D). However, other factors like AKT, ERK show no significant changes after NLGP treatment (Figures 5A–C), even after blocking them (Figures 5E,F). But, inhibition of Stat3 by Stat3-specific siRNA treatment significantly abrogated this NLGP mediated downregulation of HIF1α after 24 h (Figures 5G,H).

## Discussions

Angiogenesis plays a critical role in tumor growth by inducing VEGF secretion, which ultimately creates hypoxia and fosters tumor-promoting events and metastasis (26). Therefore, angiogenesis inhibitors specifically VEGF antagonist gained special interest in cancer management. However, these synthetic drugs generate toxicity as well as resistance. While non-toxic plant-based molecules seem more promising, detailed molecular mechanisms of action of plant based anti-angiogenic molecules are largely unknown. We reported a non-toxic immunomodulator, NLGP, having sustained effect on tumor growth restriction (27–30) and vascular normalization (21). These are primarily mediated by (i) activation of cytotoxic CD8^+^ T cells (20, 27, 29) and their interaction with CD4^+^ helper T cells (31), (ii) downregulation of immunosuppressive cytokines (IL-6, IL-10, TGFβ, etc.) (20, 24, 28, 32), (iii) decrease in suppressor cell (Treg, TAM, DC2, MDSC) accumulation and their functions (32–34), (iv) generation of central and effector memory T cells (19). However, this molecule failed to induce any direct cytocidal effect to the tumor cells (35). Here, for the first time we have shown that NLGP can directly modulate tumor cell property, which might switch the hostile TME.

A substantial reduction in uncontrolled angiogenesis is demonstrated recently after NLGP treatment in tumor bearing mice by downregulating intratumoral VEGF (21). Accordingly, the present work is initiated to understand how NLGP reduces VEGF within TME where tumor and tumor associated stromal cells including immune cells are major VEGF sources (36) and therefore could be potential targets of NLGP. To better understand the cellular target of NLGP, we looked inside a tumor tissue architecture and laser capture microdissection aided that NLGP can reduce VEGF secretion by modulating both macrophages and tumor cells. As reported earlier, NLGP significantly reduces of M2-type macrophages (24) and suppressor MDSCs (34). NLGP cannot directly kill macrophages or tumor cells thus it could modify the VEGF promoting factors like hypoxia (21). To understand the cellular and molecular mechanisms more precisely, we opt for *in vitro* culture of B16Mel, LLC, and macrophage cells, where we observed significant reduction of VEGF in tumor cells and macrophages after NLGP treatment as early as 4 h at mRNA level with more prominent result in hypoxia than normoxia, suggesting hypoxia as a major factor that NLGP targets. HIF1α a major angiogenesis inducer in tumor cells (carcinoma and melanoma) both *in vitro* and *in vivo*. Hence, those conditions known to activate HIF1α can upregulate VEGF production by recruiting other cofactors forming HIF activational complex (37). Therefore, possible mechanisms by which NLGP inhibits VEGF gene expression could include (i) Promotion of degradation of HIF1α protein, (ii) Reduction of HIF1α protein synthesis, (iii) Prevention of nuclear translocation of HIF1α, (iv) Hindrance in active HIF1α complex formation by preventing binding between HIF1α and partner(s), (v) Direct block in binding of HIF1α complex to HRE region of VEGF promoter. As we carried out our experiments in hypoxia, which stabilizes HIF1α, we did not find any changes in expression of HIF1α mRNA and protein up to 24 h with NLGP treatment, so the first possibility is obsolete. Furthermore, degradation of HIF1α protein requires obligatory presence of VHL (38), NLGP has no effect *in vivo* or *in vitro* over VHL expression. The second possibility at least for early (at 4 h) is ruled out since NLGP can only reduce HIF1α protein expression after 18 h. NLGP treatment failed to reduce the accumulation of nuclear HIF1α and can downregulate the same only after 24 h, excluding the third possibility for early VEGF downregulation. Next, we look further into the binding capacity of HIF1α with other cofactors, like Sp1, CBP, p300, and we found that NLGP prevents co-localization of HIF1α with CBP, p300 and Sp1 but not with Sp3, which can competitively target HIF1α and Sp1 binding. Sp1 and Sp3 compete for binding to the HRE region as they share more than 90% sequence homology (39–41). Consequently, we observed significant less binding of active HIF1α complex with HRE region. Moreover, this effect is more global as NLGP treatment shows downregulated expression of other HIF1α target genes (PDK1 and EPO) (data not shown).

**Figure 6:**
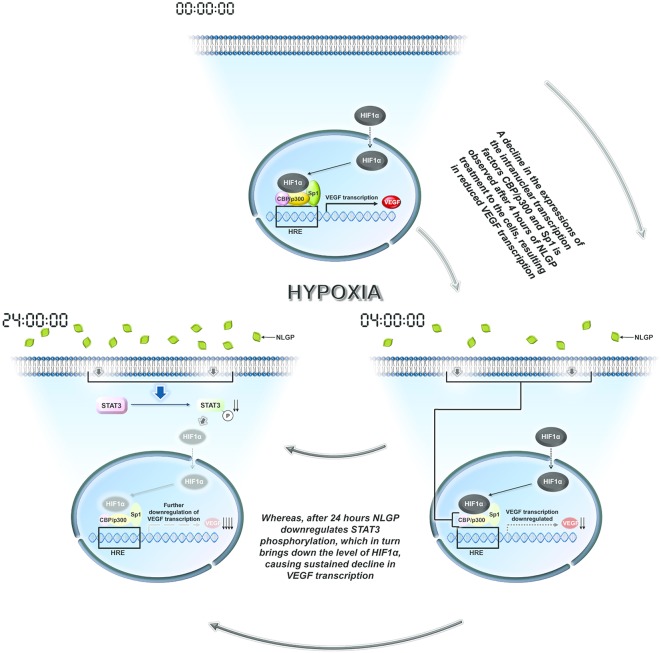
A representative pictorial of the total time events with NLGP. Schematic diagram of all overall pathway discussed. During hypoxia HIF activational complex is formed in the nucleus due to availability of stable HIF1α and binds to the HRE region of VEGF promoter to enhance VEGF transcription. When B16Mel cells were treated with NLGP under hypoxia, it modulates the system to downregulate Sp1, CBP, and p300 at 4 h but not HIF1α. This event causes less binding of the HIF activational complex to the HRE region. At 24 h it was seen that NLGP could further downregulates pStat3 expression which maintains a sustained VEGF reduction. Downregulated molecules are shaded light.

Many plant-based molecules show inhibitory effects toward VEGF mainly by modulating HIF1α protein synthesis or degradation (42–49). On the other hand, NLGP initially target and destabilizes HIF1α-HIF1β-Sp1-CBP-p300 active transcriptional complex formation at 4 h, while at late hours it downregulates HIF1α protein synthesis. Several signaling pathways are responsible for downregulation of HIF1α, like AKT, ERK, MAPK, STAT3, etc. The status of AKT and ERK phosphorylation in B16Mel cells did not show any NLGP induced changes in either normoxia or hypoxia. However, NLGP causes significant reduction in Stat3 phosphorylation under hypoxia. Consistent with our previous reports, we observed downregulation of pStat3 by NLGP and siRNA mediated knock-down of stat3, both of which mimics NLGP's effect. Recent studies suggest that HIF1α is regulated by STAT3 as the later increases the half-life of the transcription factor in both human and mouse melanoma cells (50). STAT3 can directly bind to HIF1α promoter and upregulates its expression post-translationally (51). A pictorial shown in Figure 6 demonstrates how NLGP regulates VEGF in hypoxic TME at 4 and 24 h.

A significant reduction of HIF1α-VEGF signaling axis within tumor by NLGP treatment has immense importance even from immunological perspective, where efficacy of NLGP is already proven. Moreover, effect of NLGP is dependent on CD8^+^ T cells and reduced hypoxia-and related factors may promote susceptibility of tumor cells toward T and NK cell mediated killing via induction of autophagy as well as more efficient functioning of several immune cells by downregulating CTLA4 on T cells and PDLs on tumor cells. However, here we are unable to explore how NLGP interact with macrophage or tumor cells to reduce stat3, which may eventually prevent the formation of active HIF1α-transcriptional complex. This study adds new insight into the potential mechanism of NLGP's activity besides immunomodulation which may intensify its prospective acceptance as a novel strategy for cancer management.

## Data Availability Statement

All datasets generated for this study are included in the article/Supplementary Material.

## Ethics Statement

All experiments were performed in accordance with the guidelines provided by the (Chittaranjan National Cancer Institute, Kolkata) Institutional Animal Care and Ethics Committee (Approval No. IAEC-1774/RB-7/2016/3).

## Author Contributions

AS, ABo, and RB: experimental design of the study, planning and execution, analysis of data, and manuscript preparation. AS, PN, and SD: performed majority research. ABh, NG, TG, IG, and SB: performed minor research work and helped in providing resources. ABo and RB: supervised the project and acquired funds. All authors approved the manuscript.

### Conflict of Interest

The authors declare that the research was conducted in the absence of any commercial or financial relationships that could be construed as a potential conflict of interest.
