# Supplementary material for: Neem Leaf Glycoprotein Restrains VEGF Production by Direct Modulation of HIF1α-Linked Upstream and Downstream Cascades
Source: Front Oncol. 2020 Mar 6;10:260. doi: 10.3389/fonc.2020.00260 (PMC7067891; doi:10.3389/fonc.2020.00260)
Supplement: Supplementary file 1 [file Presentation_1.pptx]

## Slide 1
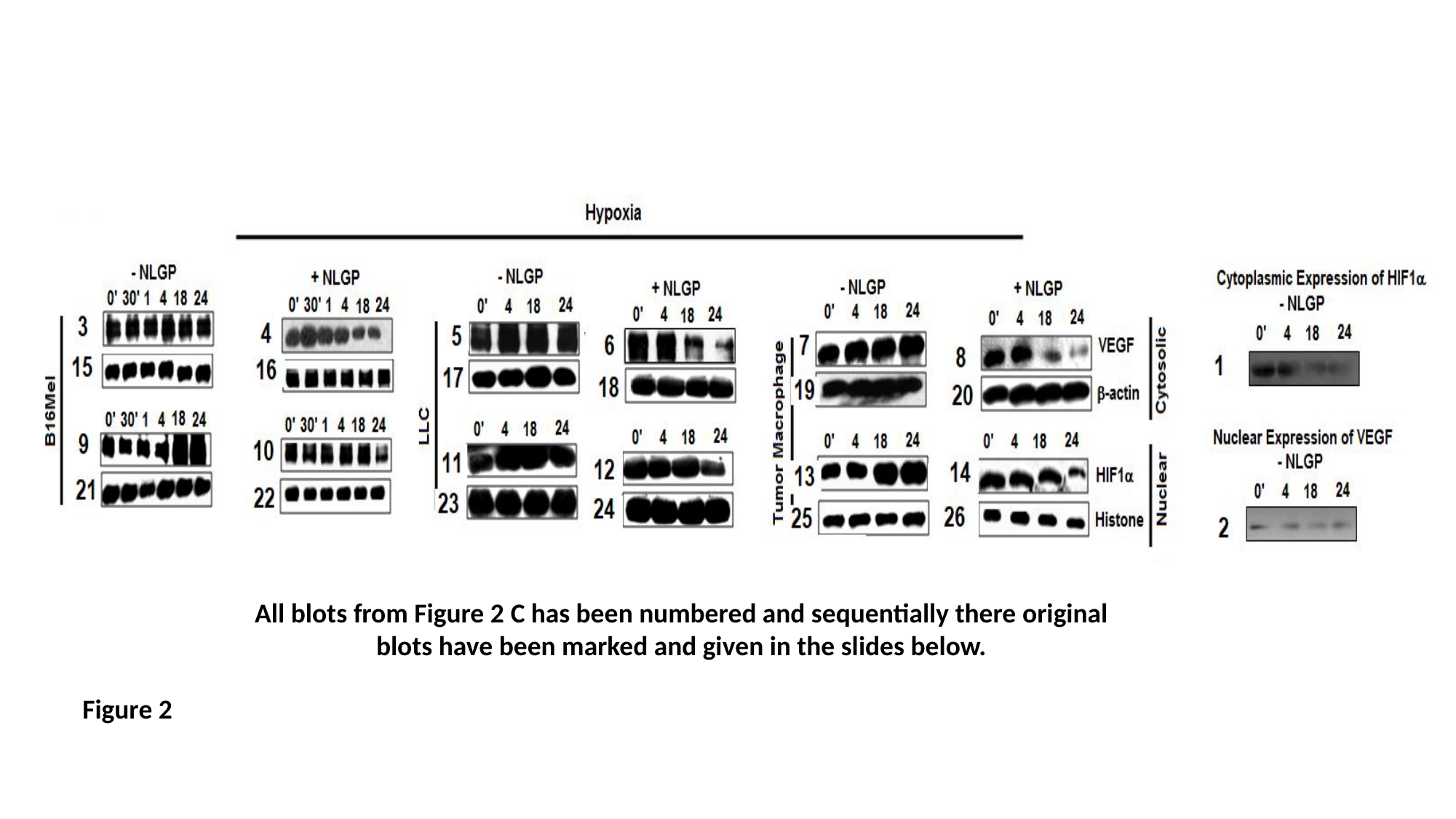

All blots from Figure 2 C has been numbered and sequentially there original blots have been marked and given in the slides below.
Figure 2

## Slide 2
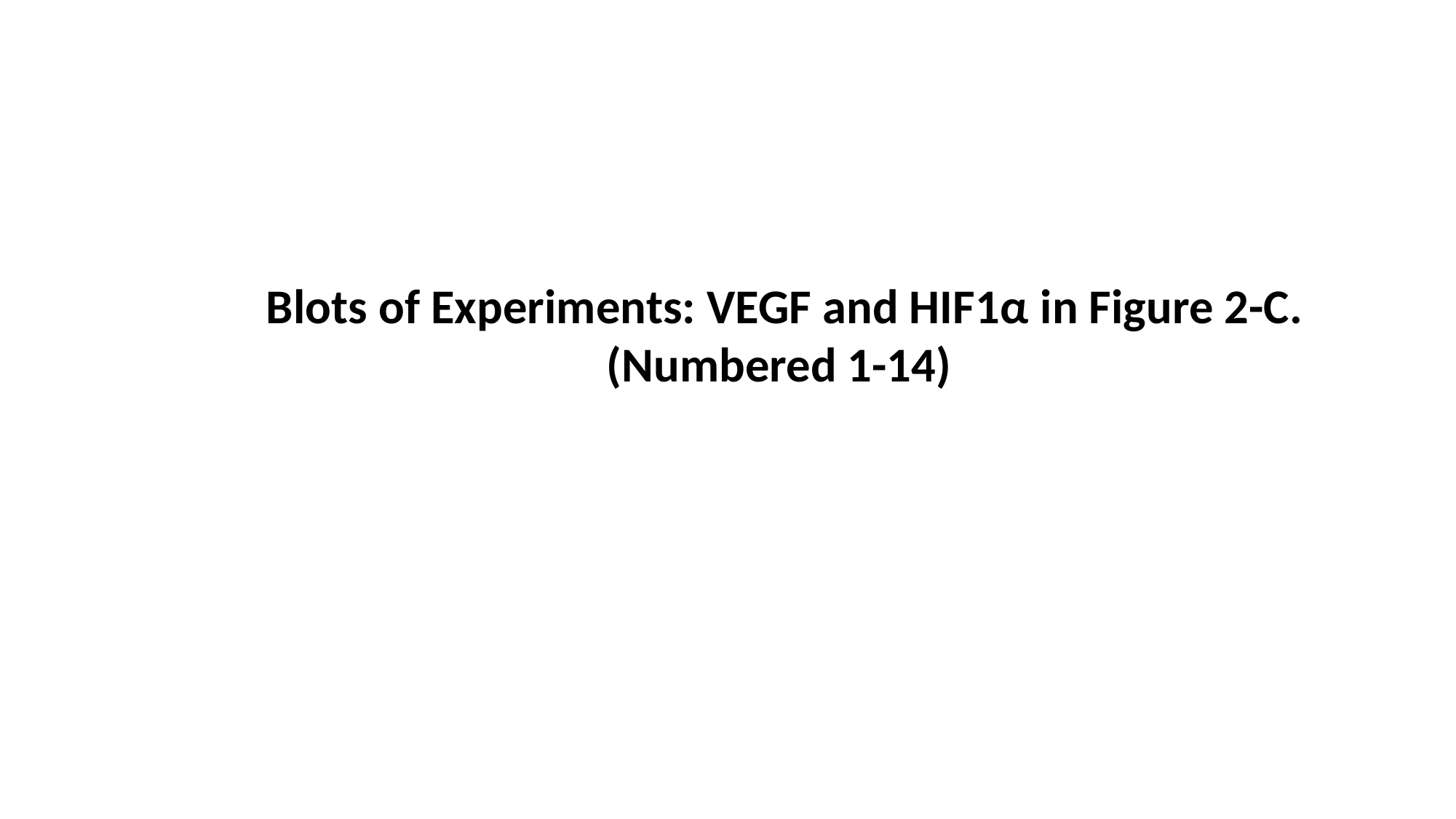

Blots of Experiments: VEGF and HIF1α in Figure 2-C. (Numbered 1-14)

## Slide 3
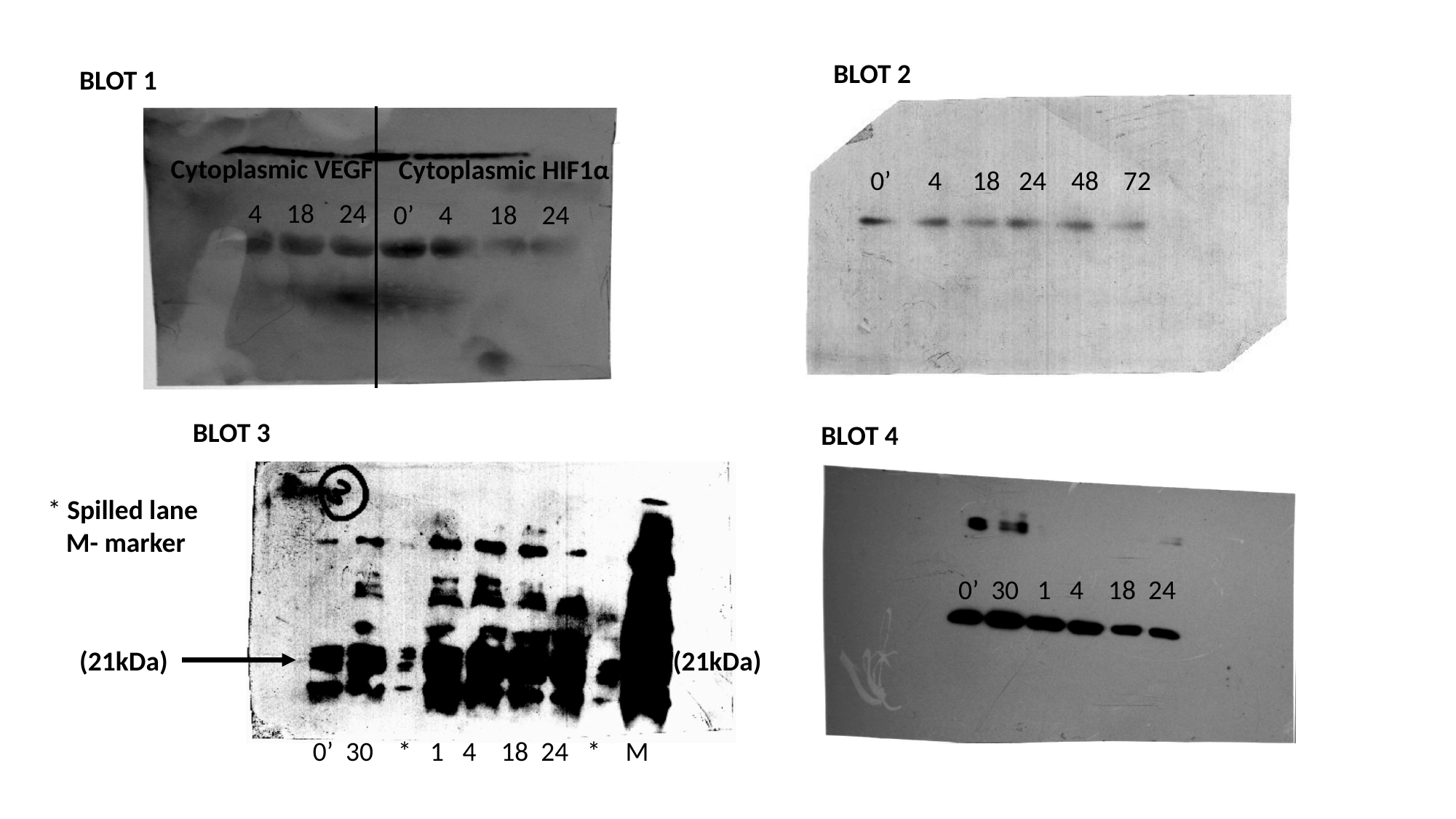

BLOT 2
BLOT 1
Cytoplasmic VEGF
Cytoplasmic HIF1α
 0’ 4 18 24 48 72
 4 18 24
 0’ 4 18 24
BLOT 3
BLOT 4
* Spilled lane
 M- marker
 0’ 30 1 4 18 24
(21kDa)
(21kDa)
 0’ 30 * 1 4 18 24 * M

## Slide 4
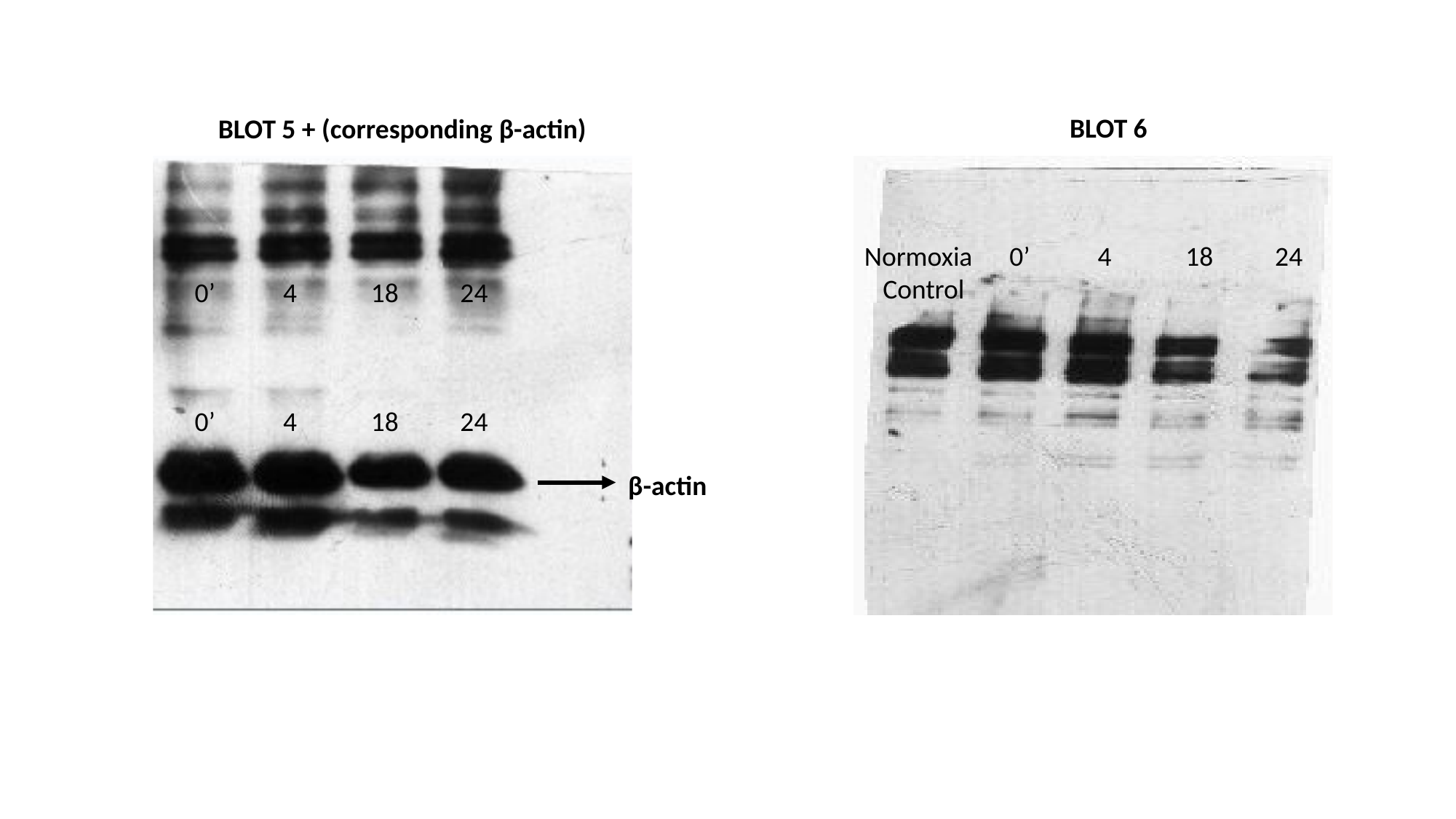

BLOT 6
BLOT 5 + (corresponding β-actin)
Normoxia 0’ 4 18 24
 Control
 0’ 4 18 24
 0’ 4 18 24
β-actin

## Slide 5
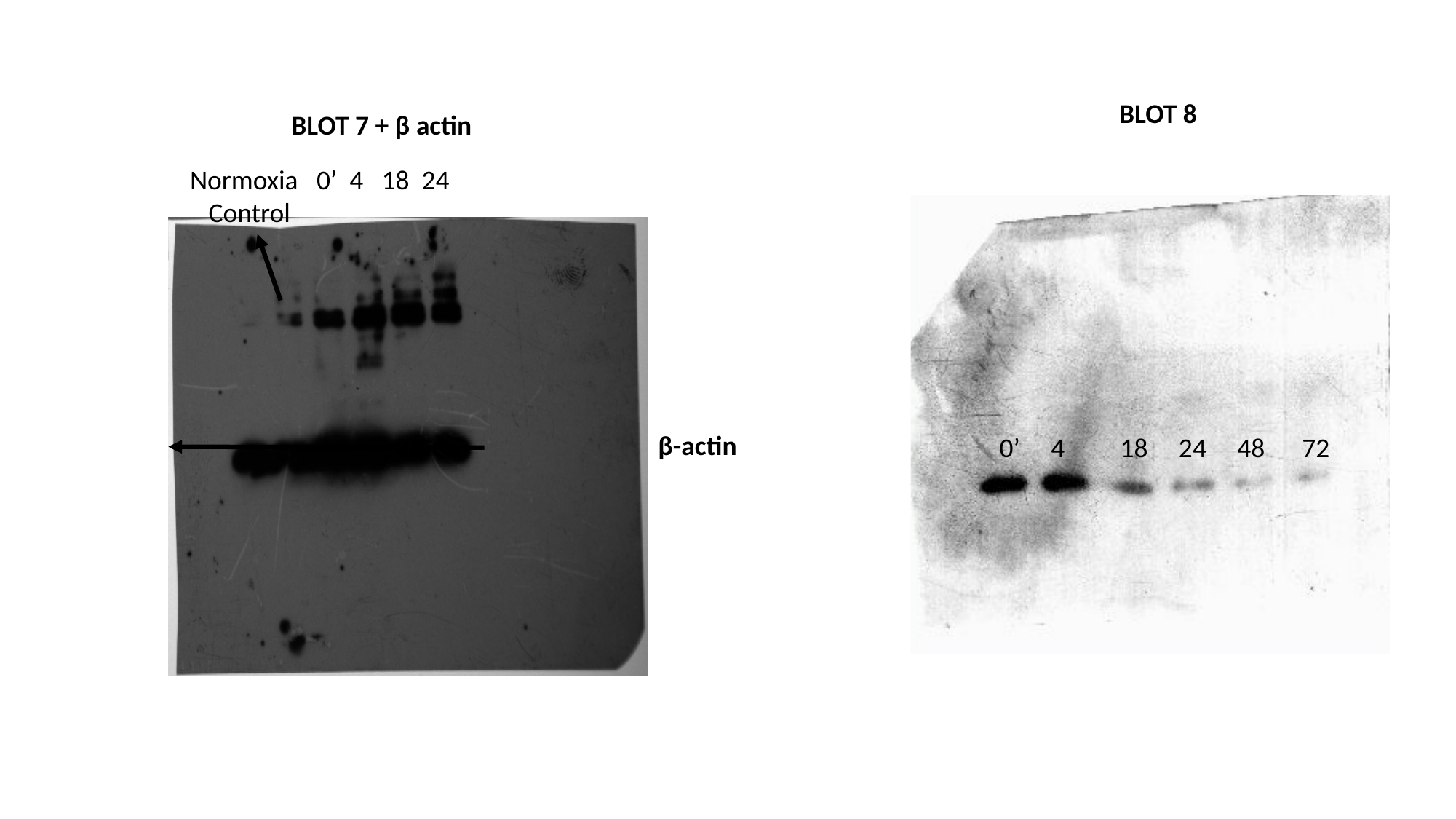

BLOT 8
BLOT 7 + β actin
Normoxia 0’ 4 18 24
 Control
β-actin
 0’ 4 18 24 48 72

## Slide 6
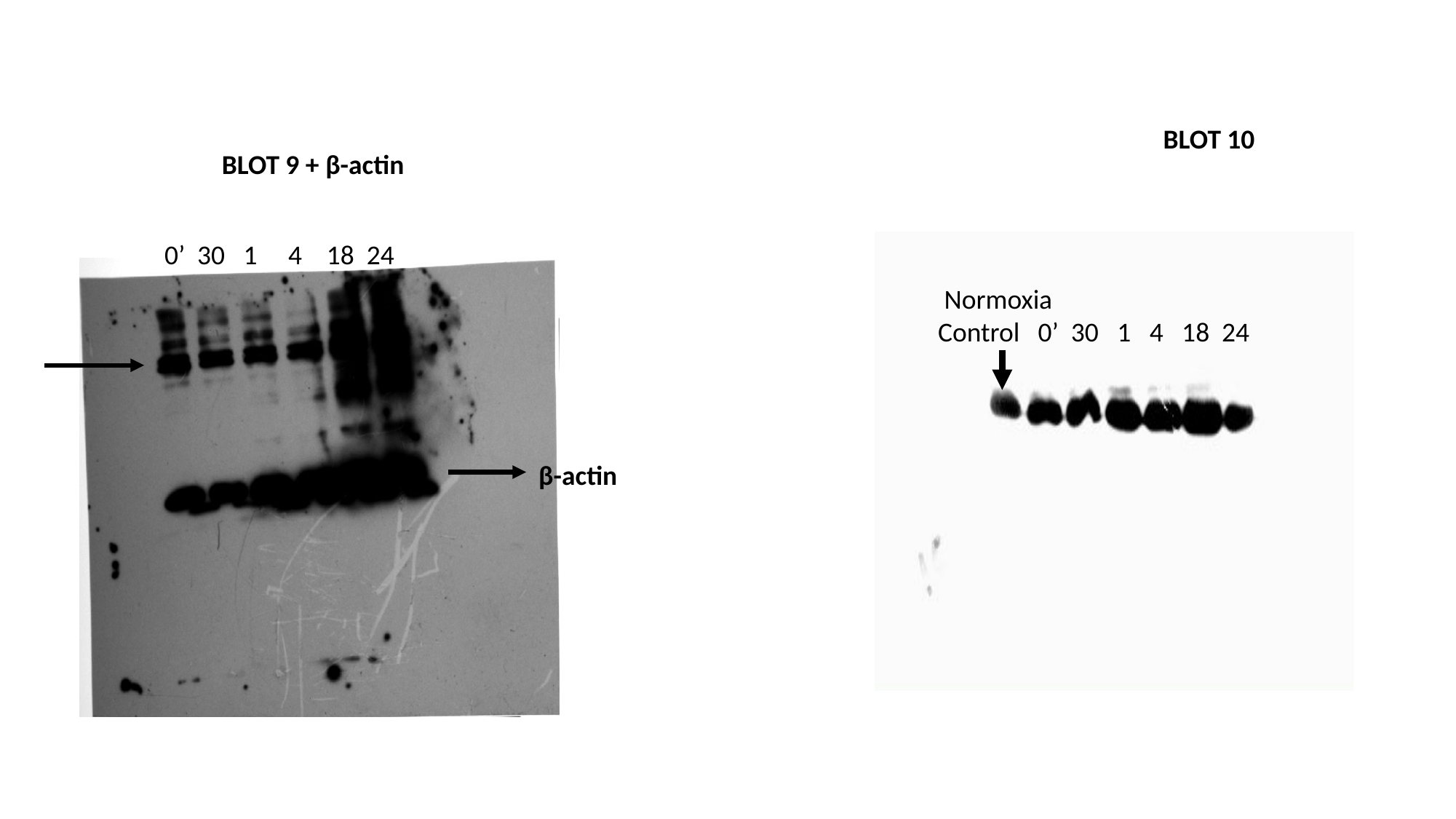

BLOT 10
BLOT 9 + β-actin
 0’ 30 1 4 18 24
 Normoxia
 Control 0’ 30 1 4 18 24
β-actin

## Slide 7
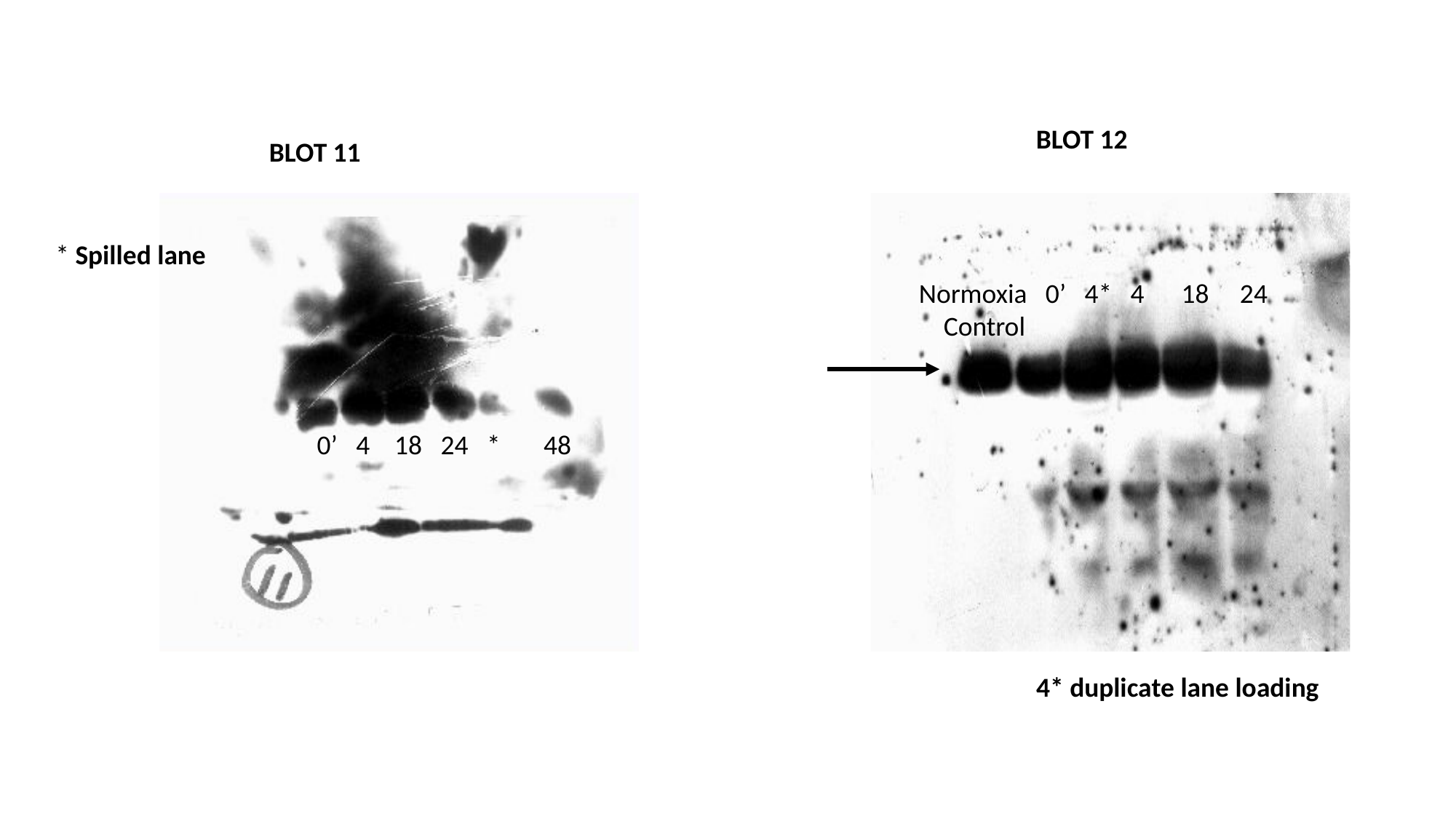

BLOT 12
BLOT 11
* Spilled lane
 Normoxia 0’ 4* 4 18 24
 Control
 0’ 4 18 24 * 48
4* duplicate lane loading

## Slide 8
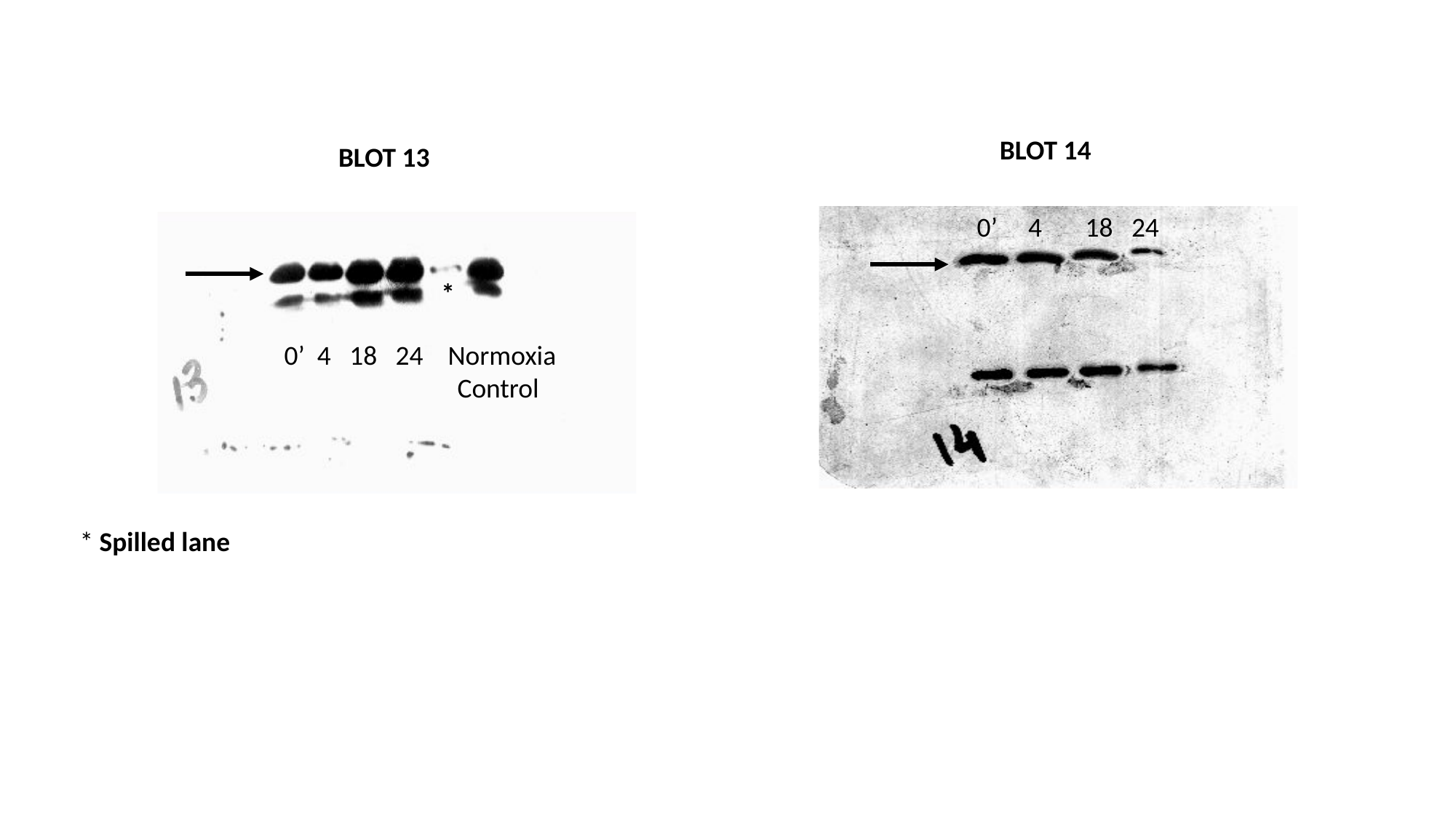

BLOT 14
BLOT 13
 0’ 4 18 24
*
 0’ 4 18 24 Normoxia
 Control
* Spilled lane

## Slide 9
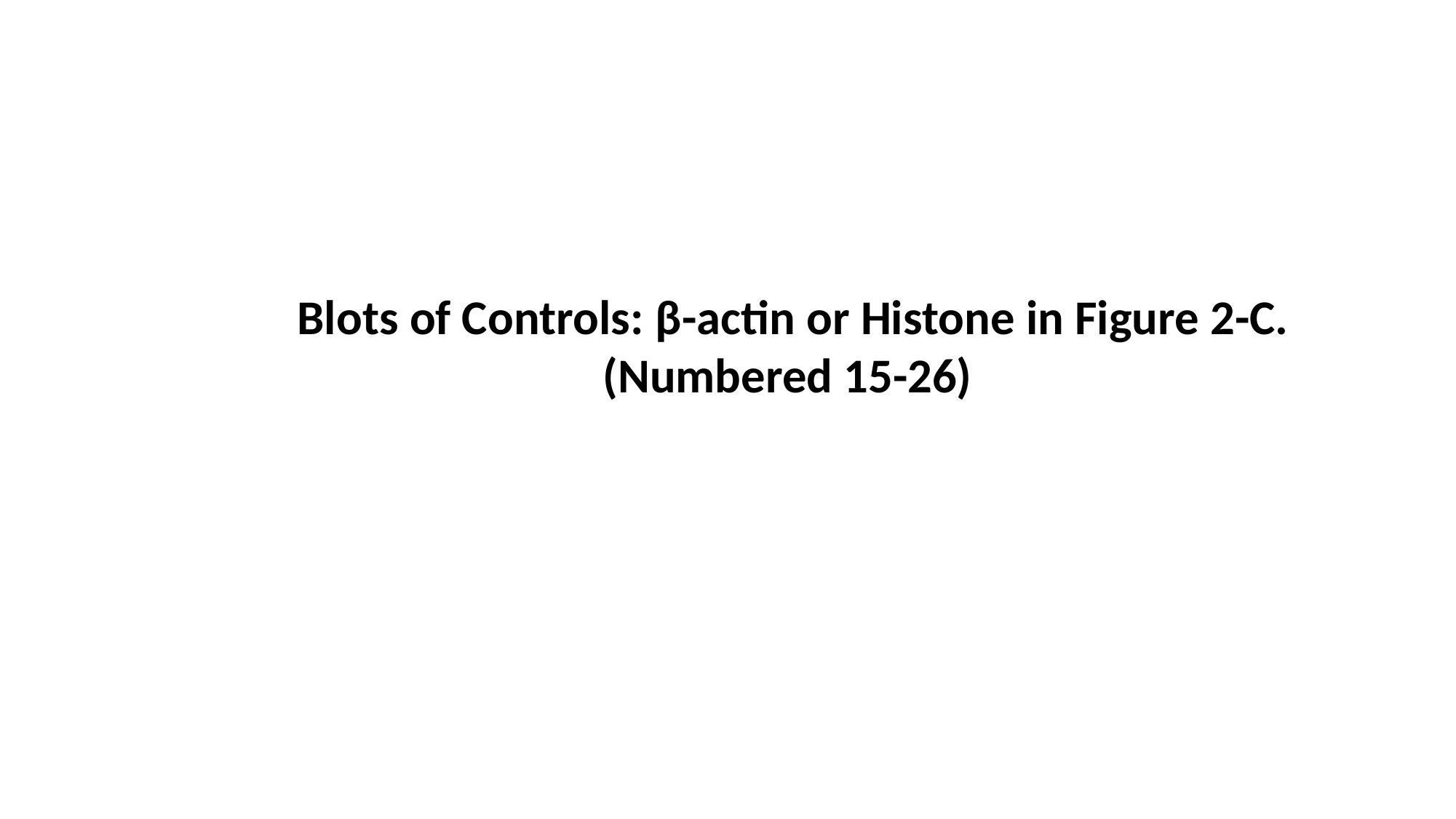

Blots of Controls: β-actin or Histone in Figure 2-C. (Numbered 15-26)

## Slide 10
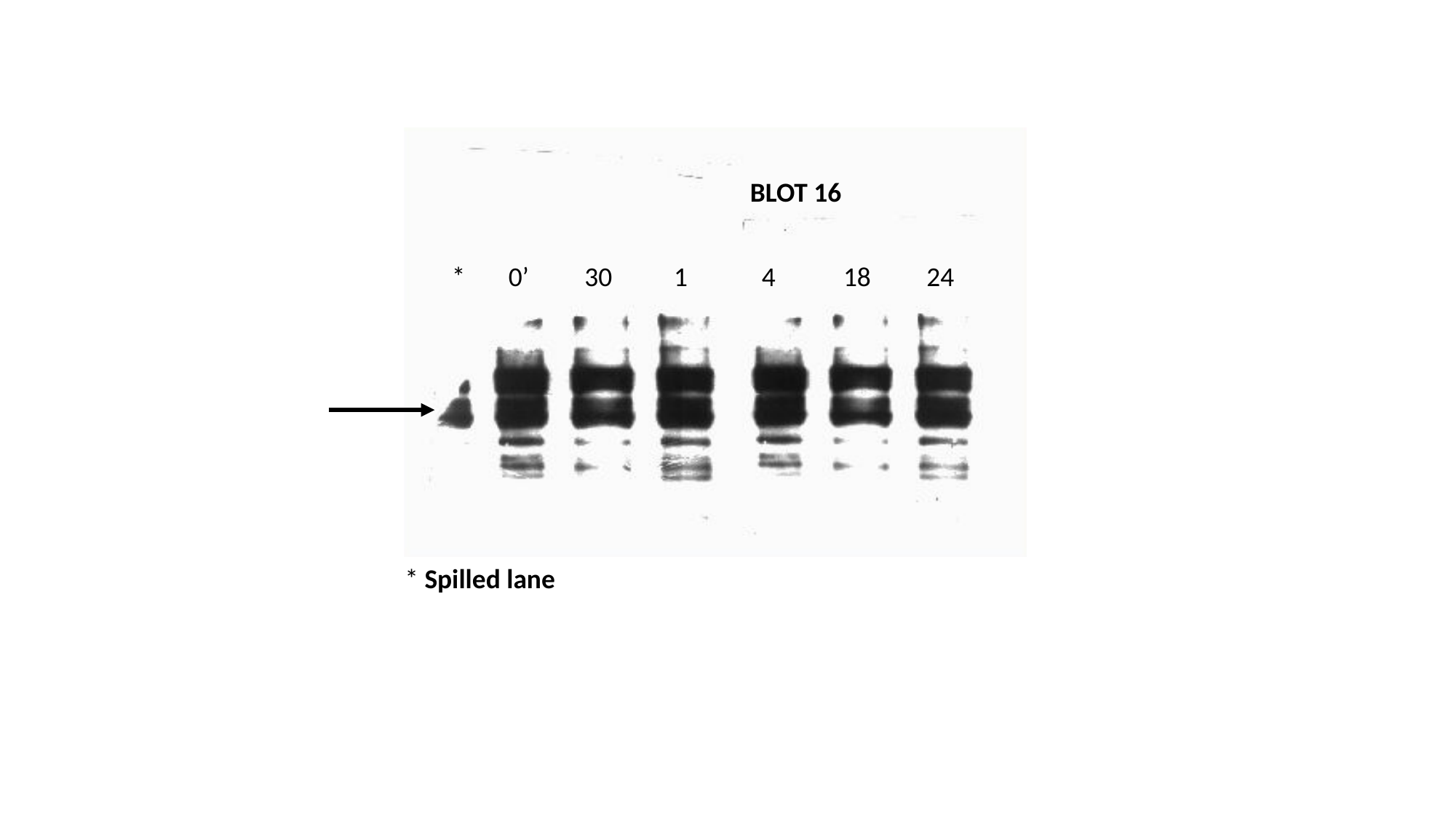

BLOT 16
 * 0’ 30 1 4 18 24
* Spilled lane

## Slide 11
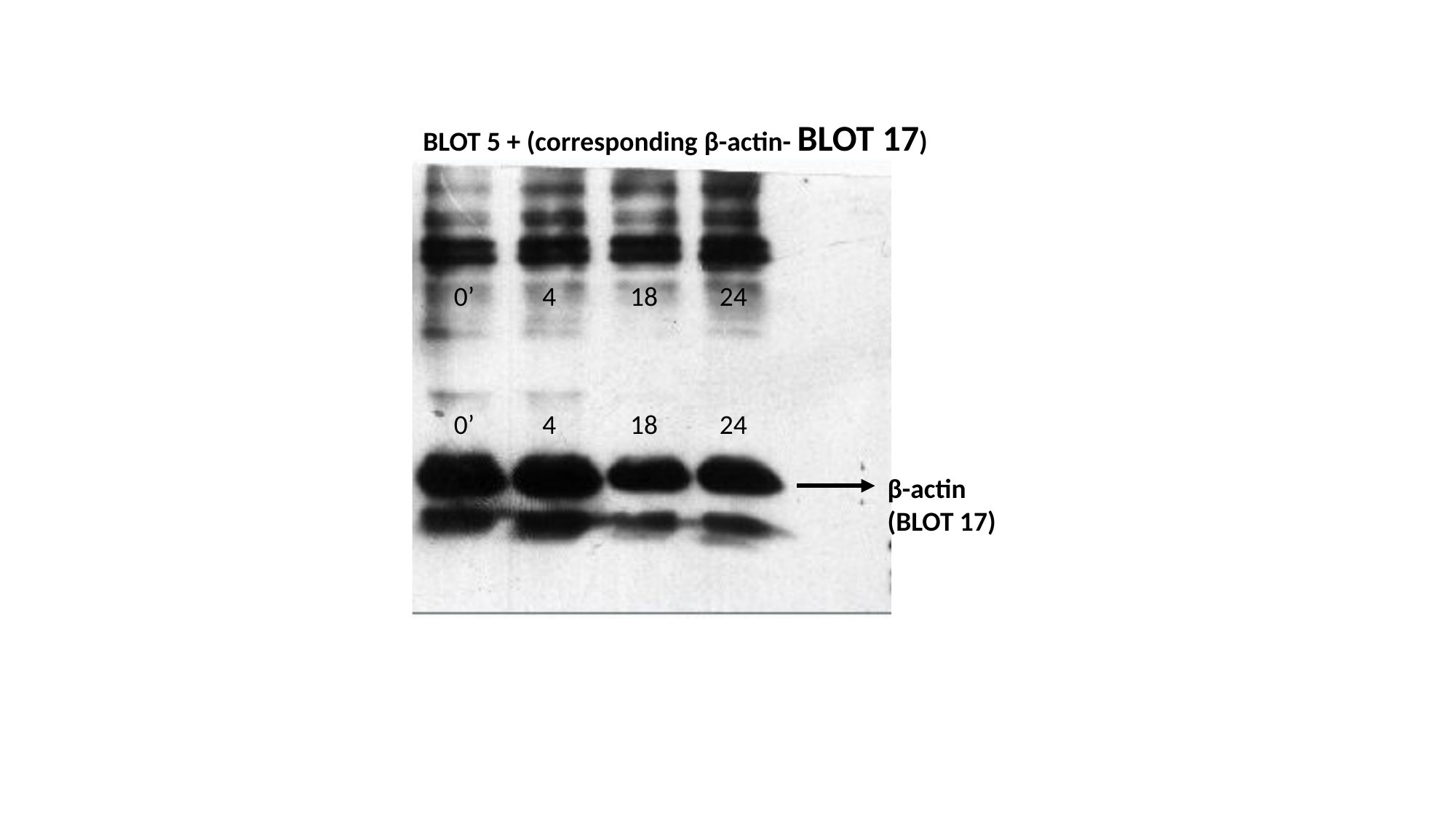

BLOT 5 + (corresponding β-actin- BLOT 17)
 0’ 4 18 24
 0’ 4 18 24
β-actin (BLOT 17)

## Slide 12
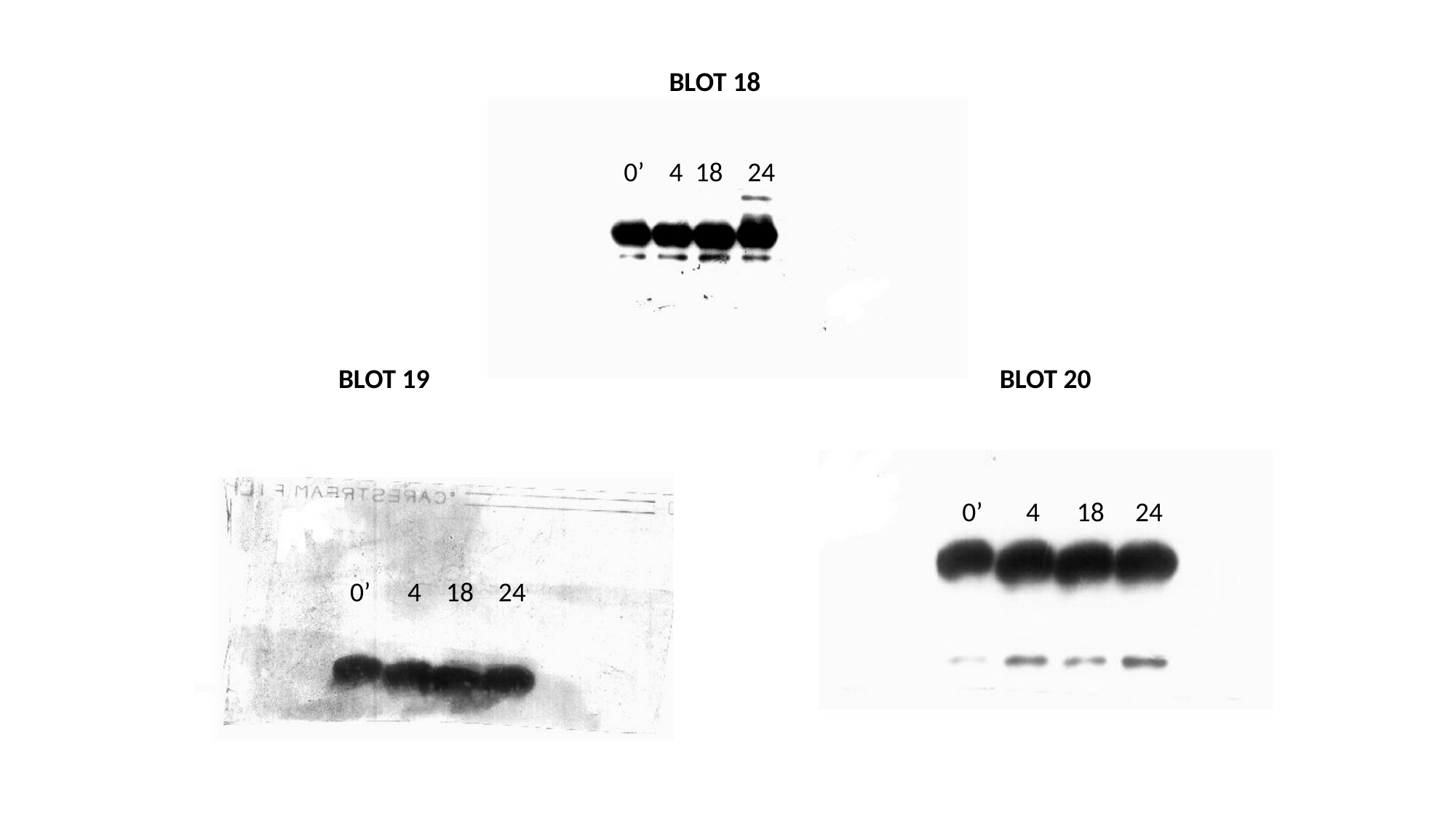

BLOT 18
 0’ 4 18 24
BLOT 19
BLOT 20
 0’ 4 18 24
 0’ 4 18 24

## Slide 13
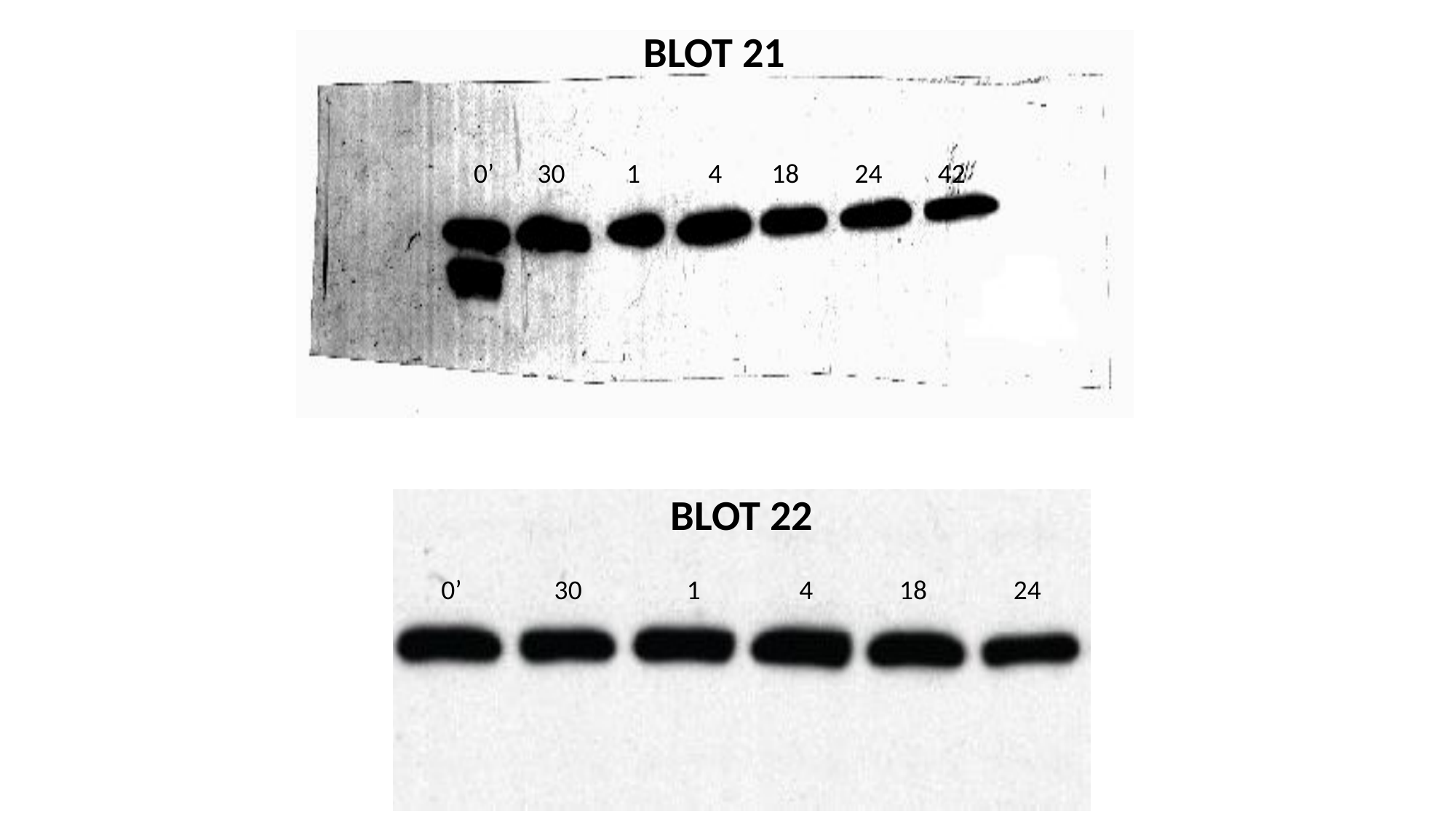

BLOT 21
 0’ 30 1 4 18 24 42
BLOT 22
 0’ 30 1 4 18 24

## Slide 14
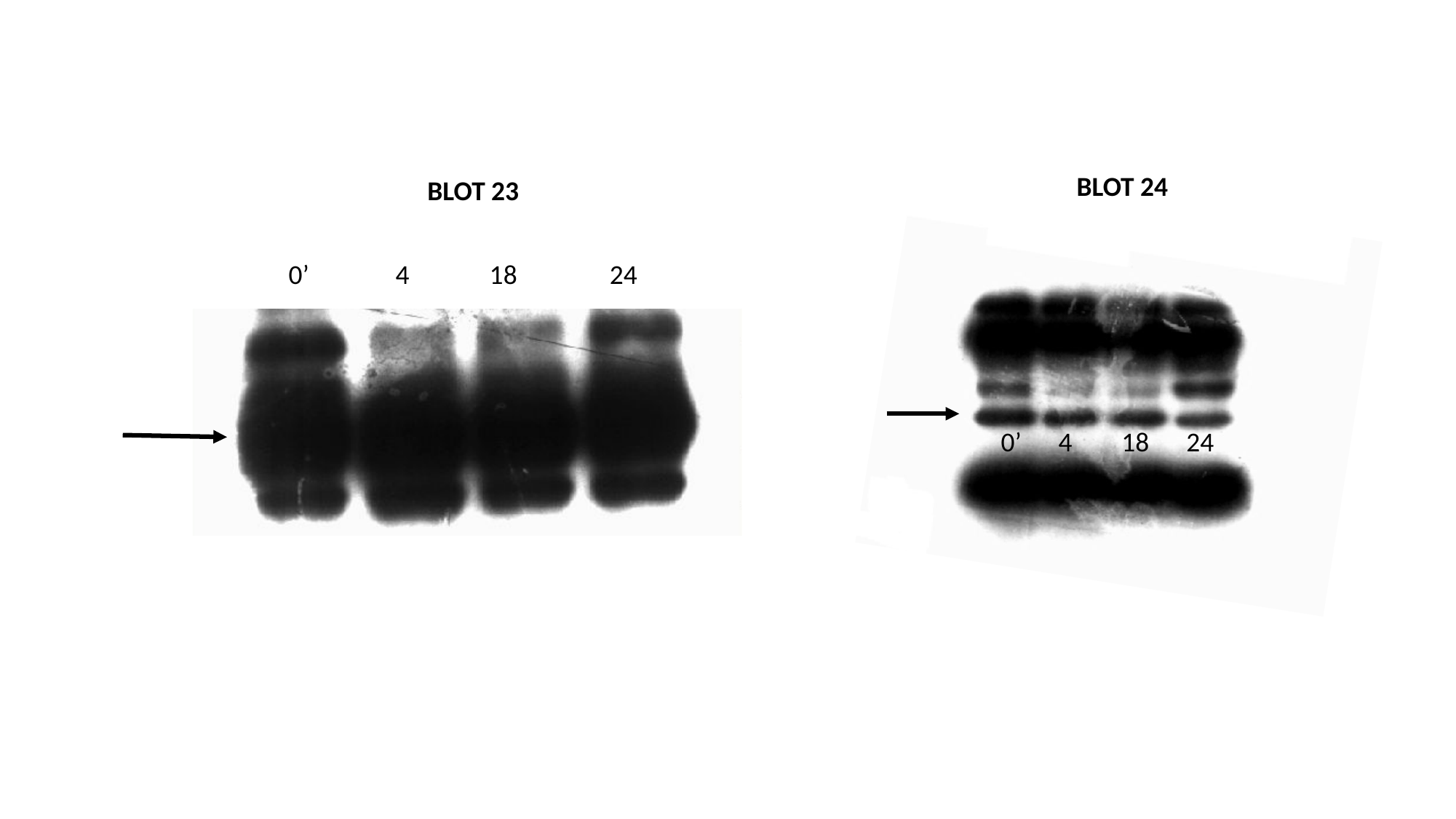

BLOT 24
BLOT 23
 0’ 4 18 24
 0’ 4 18 24

## Slide 15
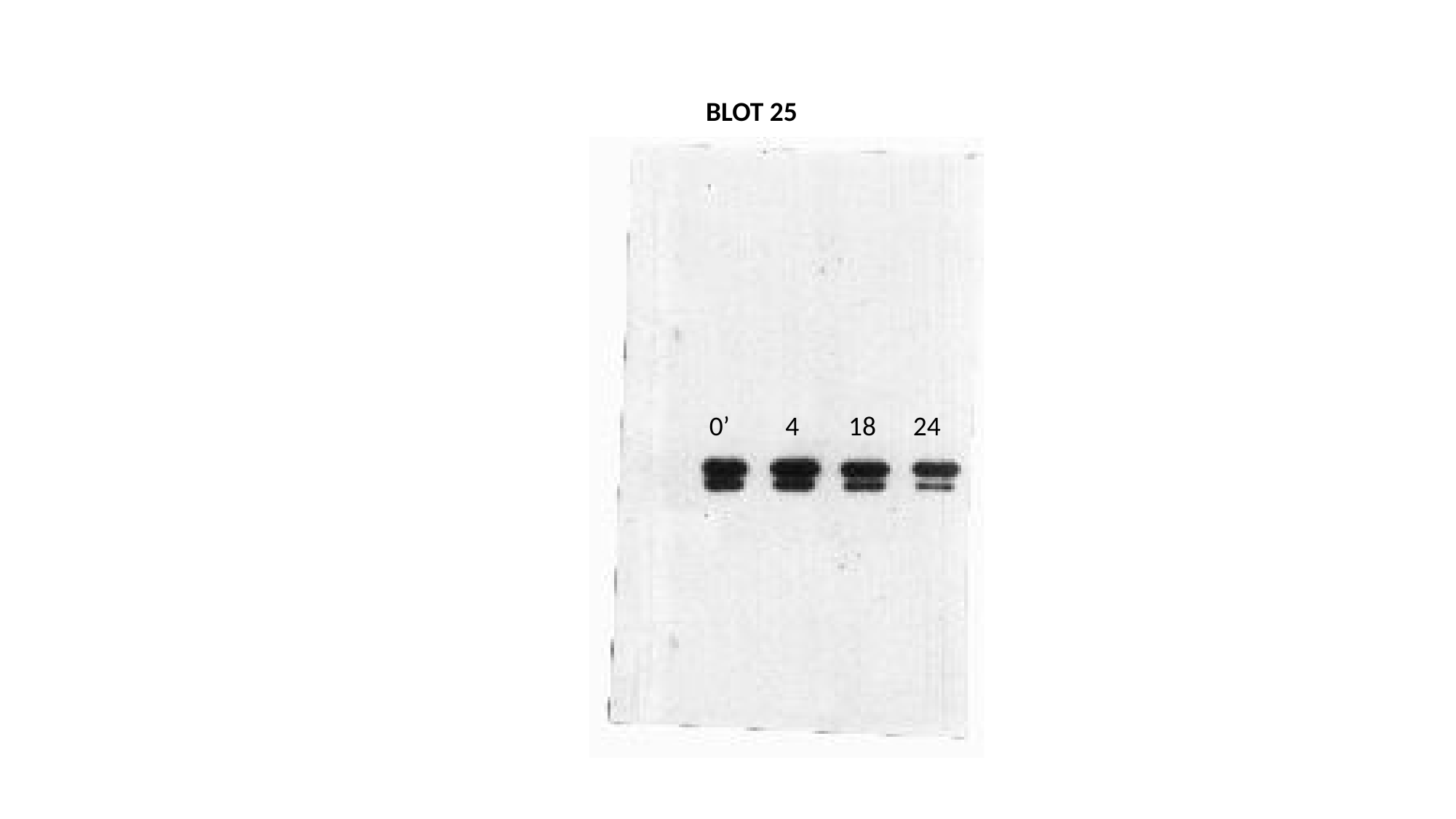

BLOT 25
 0’ 4 18 24

## Slide 16
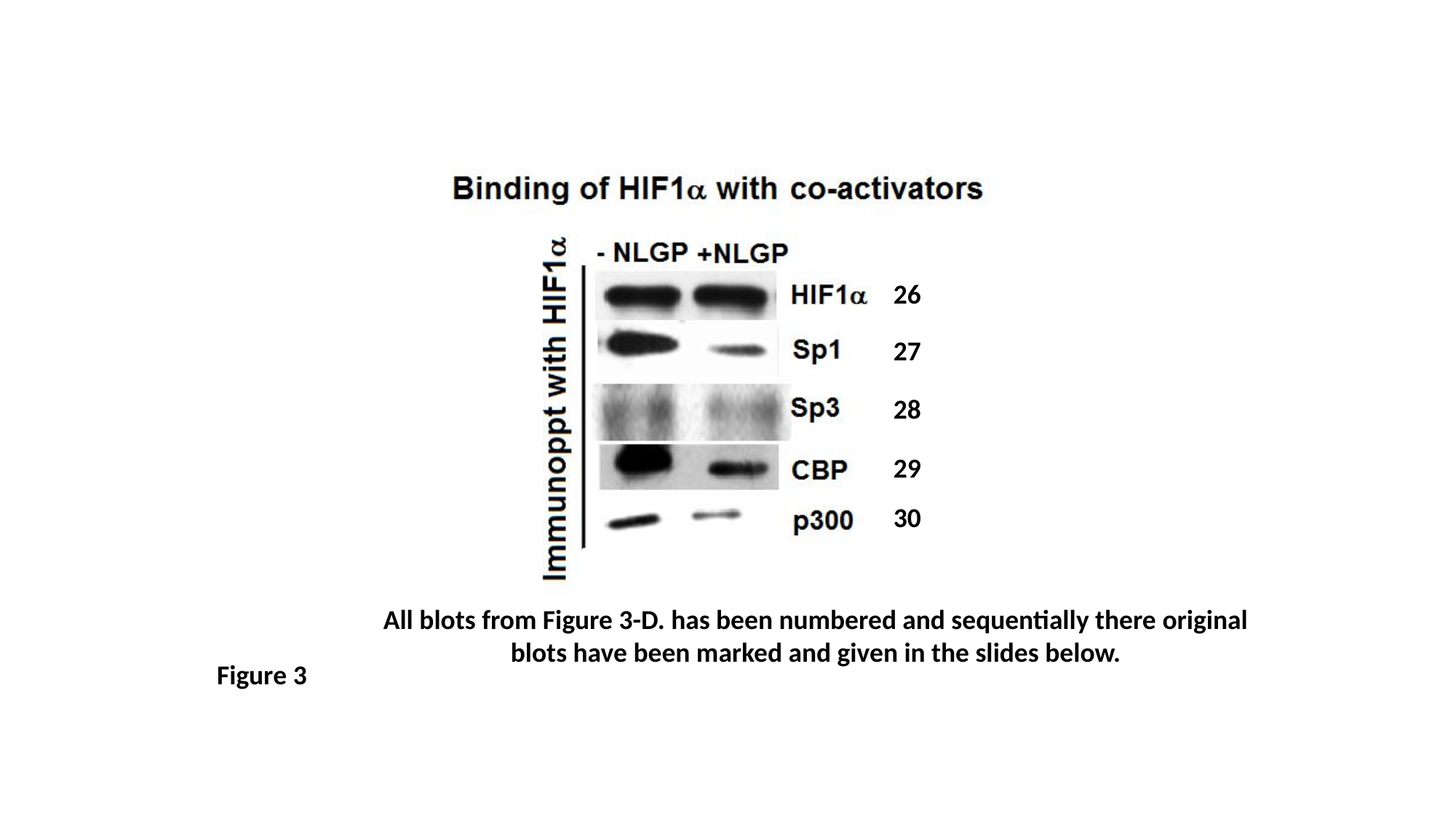

26
27
28
29
30
All blots from Figure 3-D. has been numbered and sequentially there original blots have been marked and given in the slides below.
Figure 3

## Slide 17
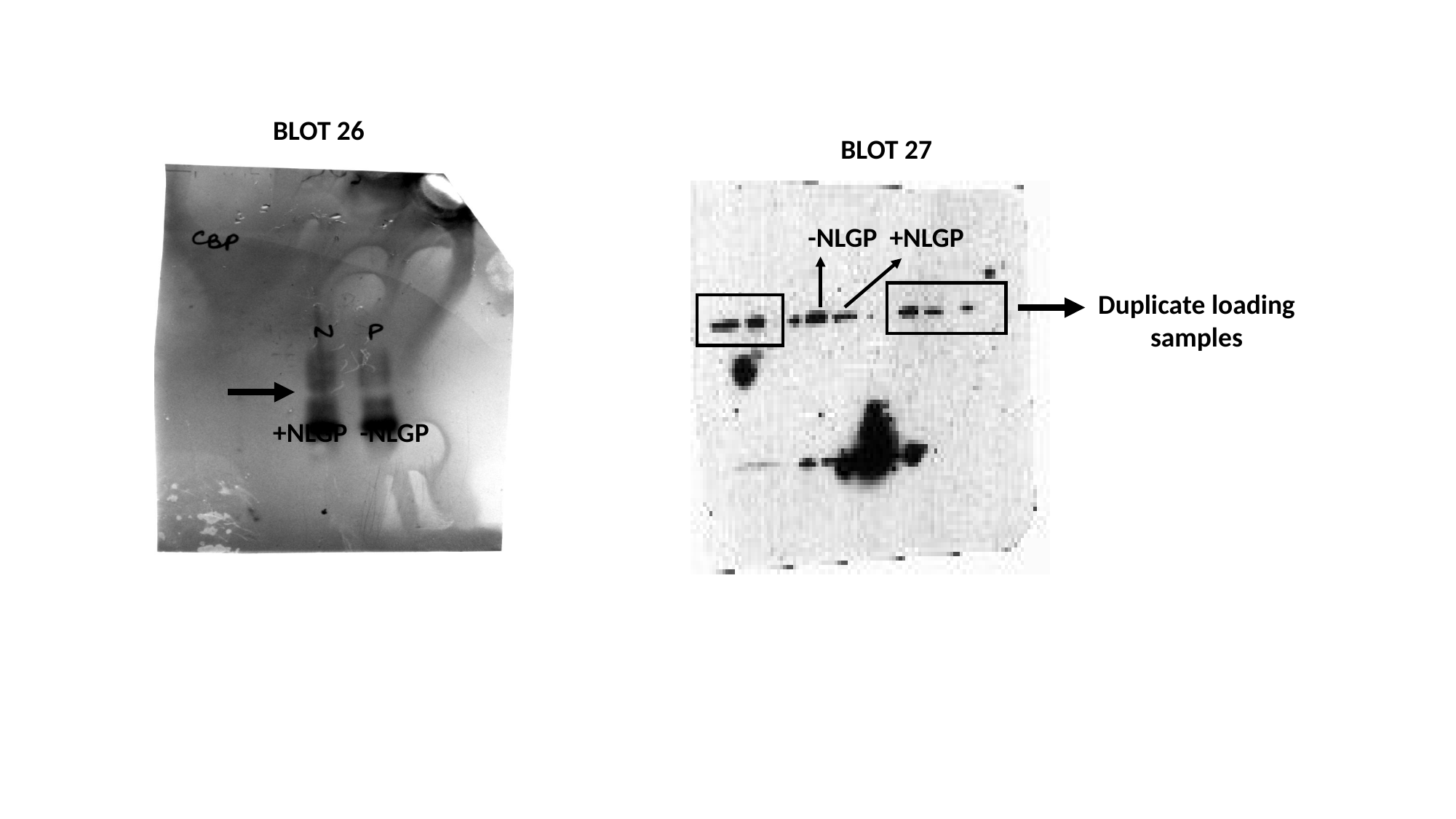

BLOT 26
BLOT 27
-NLGP +NLGP
Duplicate loading samples
+NLGP -NLGP

## Slide 18
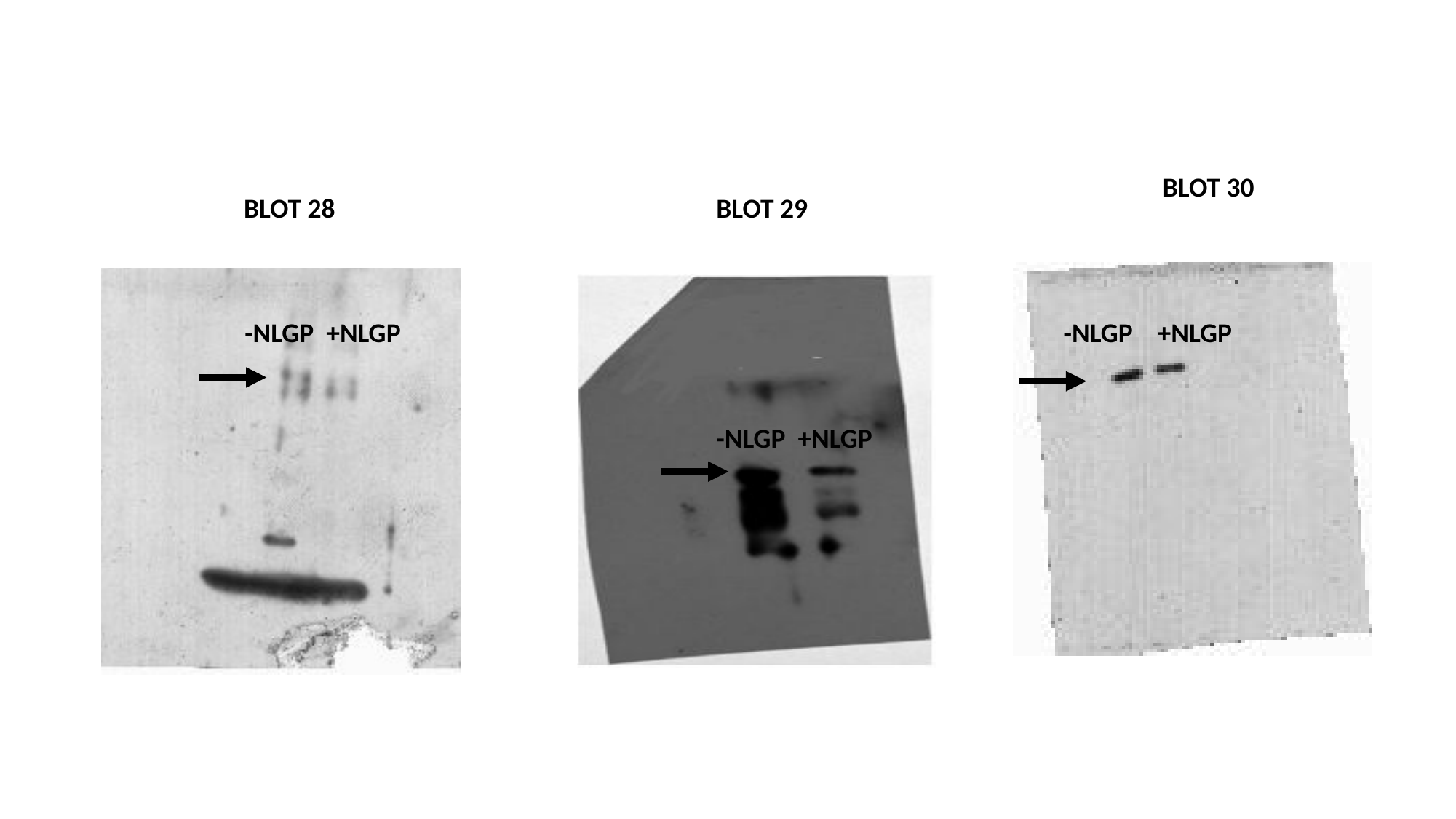

BLOT 30
BLOT 29
BLOT 28
-NLGP +NLGP
-NLGP +NLGP
-NLGP +NLGP
